# MicroRNA-Mediated Metabolic Reprograming in Renal Cancer

**DOI:** 10.3390/cancers11121825

**Published:** 2019-11-20

**Authors:** Joanna Bogusławska, Piotr Popławski, Saleh Alseekh, Marta Koblowska, Roksana Iwanicka-Nowicka, Beata Rybicka, Hanna Kędzierska, Katarzyna Głuchowska, Karolina Hanusek, Zbigniew Tański, Alisdair R. Fernie, Agnieszka Piekiełko-Witkowska

**Affiliations:** 1 Department of Biochemistry and Molecular Biology, Centre of Postgraduate Medical Education, ul. Marymoncka 99/103, 01-813 Warsaw, Poland; joanna.boguslawska@cmkp.edu.pl (J.B.); piotr.poplawski@cmkp.edu.pl (P.P.); beata.rybicka@cmkp.edu.pl (B.R.); h.kedzierska@cent.uw.edu.pl (H.K.); katarzyna.rodzik@cmkp.edu.pl (K.G.); karolina.hanusek@cmkp.edu.pl (K.H.); 2 Max-Planck Institute of Molecular Plant Physiology, 14476 Potsdam-Golm, Germany; Alseekh@mpimp-golm.mpg.de (S.A.); Fernie@mpimp-golm.mpg.de (A.R.F.); 3 Center for Plant Systems Biology and Biotechnology, 4000 Plovdiv, Bulgaria; 4 Laboratory of Systems Biology, Faculty of Biology, University of Warsaw, 02-106 Warsaw, Poland; marta@ibb.waw.pl (M.K.); roxana@ibb.waw.pl (R.I.-N.); 5 Laboratory for Microarray Analysis, Institute of Biochemistry and Biophysics, Polish Academy of Sciences, 02-106 Warsaw, Poland; 6 Masovian Specialist Hospital in Ostroleka, 07-410 Ostroleka, Poland; tanska@interia.pl

**Keywords:** renal cell cancer, microRNA, metabolome, proliferation, PPP, pentose phosphate pathway, TCA cycle, miR-155-5p, miR-146a-5p, TCGA

## Abstract

Metabolic reprogramming is one of the hallmarks of renal cell cancer (RCC). We hypothesized that altered metabolism of RCC cells results from dysregulation of microRNAs targeting metabolically relevant genes. Combined large-scale transcriptomic and metabolic analysis of RCC patients tissue samples revealed a group of microRNAs that contribute to metabolic reprogramming in RCC. miRNAs expressions correlated with their predicted target genes and with gas chromatography-mass spectrometry (GC-MS) metabolome profiles of RCC tumors. Assays performed in RCC-derived cell lines showed that miR-146a-5p and miR-155-5p targeted genes of PPP (the pentose phosphate pathway) (*G6PD* and *TKT*), the TCA (tricarboxylic acid cycle) cycle (*SUCLG2*), and arginine metabolism (*GATM*), respectively. miR-106b-5p and miR-122-5p regulated the NFAT5 osmoregulatory transcription factor. Altered expressions of G6PD, TKT, SUCLG2, GATM, miR-106b-5p, miR-155-5p, and miR-342-3p correlated with poor survival of RCC patients. miR-106b-5p, miR-146a-5p, and miR-342-3p stimulated proliferation of RCC cells. The analysis involving >6000 patients revealed that miR-34a-5p, miR-106b-5p, miR-146a-5p, and miR-155-5p are PanCancer metabomiRs possibly involved in global regulation of cancer metabolism. In conclusion, we found that microRNAs upregulated in renal cancer contribute to disturbed expression of key genes involved in the regulation of RCC metabolome. miR-146a-5p and miR-155-5p emerge as a key “metabomiRs” that target genes of crucial metabolic pathways (PPP (the pentose phosphate pathway), TCA cycle, and arginine metabolism).

## 1. Introduction

Renal cell cancer (RCC) is the most common subtype of kidney malignancies, affecting 300,000 people annually worldwide [1]. In approximately 25–30% of patients, metastasis is present at diagnosis, while a further 25% of patients develop metastases at later stages of the disease. Metastatic RCC (mRCC) is persistently difficult for treatment. Current therapeutic options include tyrosine kinase receptors inhibitors (TKIs), inhibitors of the mTOR (the mammalian target of rapamycin) pathway, or recently introduced inhibitors of immune checkpoints. All these treatments, however, prolong patients’ life by only up to two years [2].

Recent studies provided strong evidence that aberrant cellular metabolism contributes to development and progression of RCC. Similar to all cancers, RCC is characterized by increased consumption of glucose with simultaneous enhanced production of lactate under normal oxygen supply (the Warburg effect). The other metabolic features of RCC include alterations in the TCA (the tricarboxylic acid cycle) cycle and the pentose-phosphate pathway (PPP) as well as the metabolism of amino acids and fatty acids [3]. In our previous study we found that disturbances in the metabolism of succinate, beta-alanine, purines, glucose, and *myo*-inositol are linked with poor survival of RCC patients [4]. Remarkably, apart from changes in levels of intracellular metabolites in RCC tumors, we found significant alterations in expressions of genes encoding key metabolic pathways. The causes of these alterations remain unknown.

In the current study, we hypothesized that disturbed expression of metabolic genes in RCC could be caused by microRNAs (miRs). These short, non-coding RNAs interact with microRNA response elements (MREs) located in 3′UTRs of target transcripts and either trigger their degradation or attenuate translation, thereby contributing to the regulation of gene expression. microRNAs influence cancer development and progression by changing the expressions of oncogenes and tumor suppressors as well as genes involved in key signaling pathways. Remarkably, one microRNA can regulate multiple target genes, while one gene can be commonly regulated by several microRNAs [5]. We and others showed that disturbed expression of microRNAs in renal cancer contributes to altered expression of genes regulating proliferation, migration, invasion, and apoptosis [6,7].

Here, we hypothesized that altered expression of genes involved in metabolic regulation in RCC could result from dysregulation of their targeting microRNAs. We verified our hypothesis by comprehensively analyzing expressions of nearly 100 microRNAs predicted to target altered metabolic genes in a large group of RCC patients, in order to identify and validate miRNAs that can act as regulators of the RCC metabolome. Remarkably, we show that metabolically relevant microRNAs affect proliferation of the RCC cells and contribute to the poor survival of RCC patients. To our knowledge, this is the first study addressing the role of microRNAs in global regulation of genes affecting renal cancer metabolome.

## 2. Results

### 2.1. The Expression of miRs Predicted to Target Metabolic Genes Is Altered in Renal Tumors

**Figure 1:**
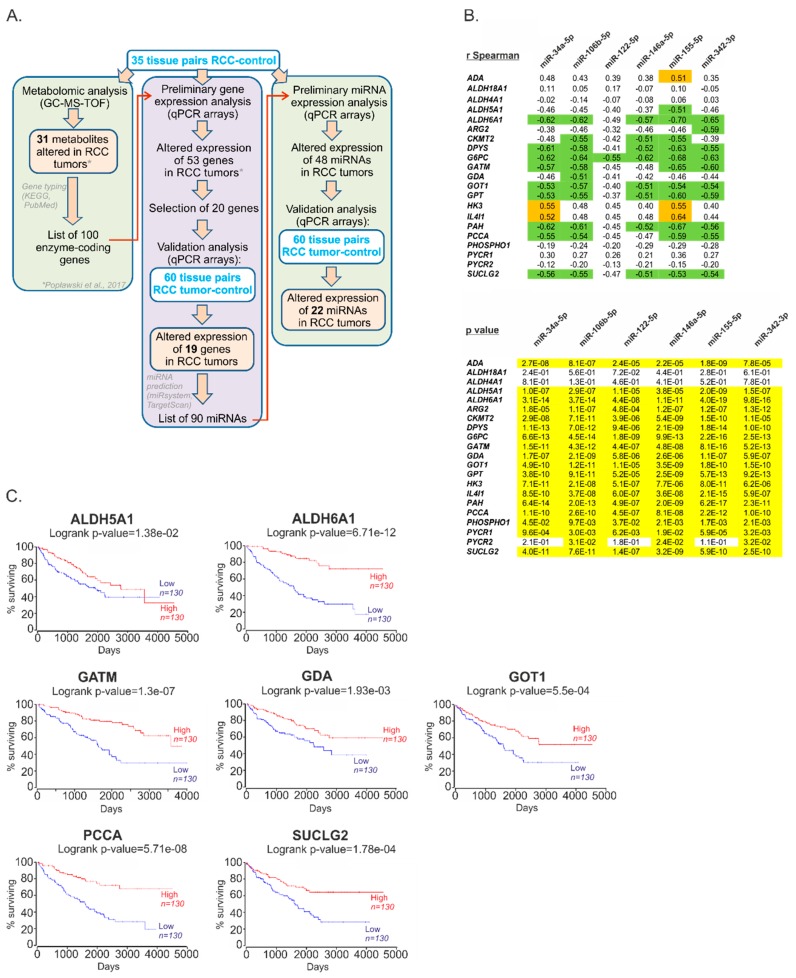
The expressions of microRNAs in relation to their predicted metabolically relevant gene targets. (**A**) The scheme of analysis of miRNAs predicted to regulated RCC metabolome. (**B**) Correlations between the expressions of metabolic genes and their predicted regulatory microRNAs, selected for functional analysis. Upper panel shows correlation coefficients. Green: r Spearman < −0.5; orange: r Spearman > 0.5. Lower panel: *p* values. Yellow: *p* < 0.05. Full data of correlation analysis are given in Table S3. *N* = 60 of RCC tumor samples and *n* = 60 of control tissue samples. (**C**) Altered expression of metabolic genes correlates with poor survival of RCC patients. Kaplan–Meier plots were generated using OncoLnc tool and KIRC (Kidney Renal Clear Cell Carcinoma) cohort of TCGA (The Cancer Genome Atlas) data. Patients were classified into Low and High expression groups basing on median mRNA expression (the expression profiles in two groups of patients are given in Figure S1). *N* = 260.

In our previous study, we identified a group of genes encoding metabolic enzymes for which altered expression was associated with changed metabolic profiles of RCC tumors [4]. Here, to validate the results of that study, we selected 20 genes based on their possible effects on patient survival, the number of predicted targeting miRNAs, and the fold changes in their expression (Table S1), and analyzed their expression in an independent group of 60 RCC-control tissue pairs (Figure 1A). This analysis confirmed altered expression of 19 genes encoding enzymes involved in the regulation of RCC metabolome (Table 1). 

Based on the results of bioinformatic analysis and the selection criteria described in the Methods Section and File S1, we selected 90 microRNAs (Table S2) predicted to regulate 19 metabolic genes and analyzed their expression in 35 matched-pairs of ccRCC (clear cell Renal Cell Carcinoma) tumors and non-tumorous kidney samples. The expression of 48 microRNAs was statistically significantly different in RCC tumors when compared to controls (*p* < 0.05; threshold of expression change: 30%) (Table S2). Next, we performed validation analysis, using an independent group of 60 matched-pairs of ccRCC tumors and control samples, and confirmed altered expression of 22 microRNAs (Table 1). microRNAs for which expression was most increased included: miR-122-5p (+107.7-fold), miR-210-3p (+10.2-fold) and miR-34a-5p (+3.1-fold).

Since the negative correlation between expression of miRNAs and target genes is a potential indicator of their functional association [8], we next checked whether the expressions of miRNAs correlated with the expressions of the metabolic genes. To this end, we constructed correlation matrix (Table S3) and searched for miRNAs of which expressions correlated with the highest number of target genes. This analysis revealed that top microRNAs for which expressions negatively correlated with genes expressions (r Spearman < −0.5, *p* < 0.05) included miR-34a-5p (9 correlating genes), miR-106b-5p (11 correlating genes), miR-146a-5p (8 correlating genes), miR-155-5p (11 correlating genes), and miR-342-3p (10 correlating genes). These five miRNAs were next selected for functional analysis of their impact on RCC cells. In addition, we also selected miR-122-5p, which was the top upregulated miRNA in RCC tumors. The correlations between miR-122-5p and metabolic genes were weaker, but still statistically significant (r Spearman = −0.35 to −0.49, *p* < 0.05) (Figure 1B). Remarkably, altered expression of all metabolic genes, predicted as targets of the selected miRNAs, correlated with poor survival of RCC patients, suggesting their potential link with the progression of the disease (Figure 1C).

**Figure 2:**
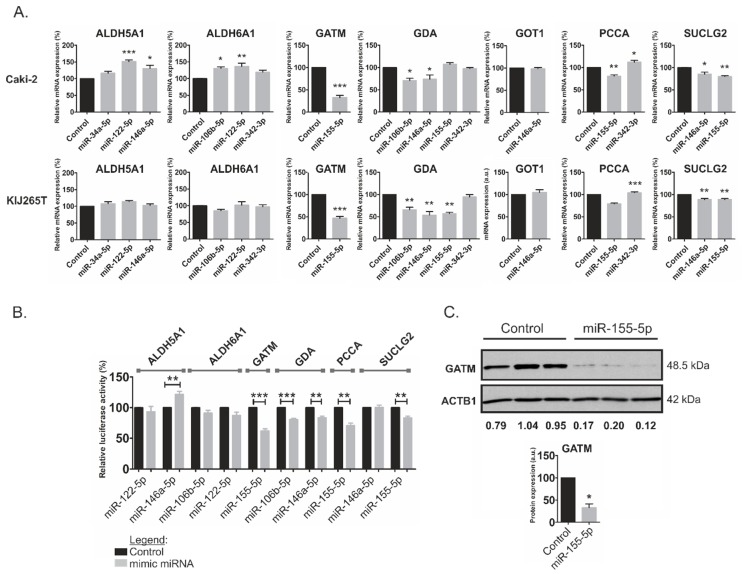
miRNA-mediated regulation of expressions of metabolically relevant genes. (**A**) The effects of miRNAs on mRNA expressions of metabolic genes predicted as potential miRNAs’ targets. Caki-2 and KIJ265T cell lines were transfected using miRNA mimics or non-targeting scrambled control oligonucleotides and expression of target genes was evaluated using qPCR (quantitative real-time PCR). The plots show results of three independent biological experiments (exception: *GDA* expression in KIJ265T cells): for most miRNAs (except for miR-106b-5p) results of two independent experiments are shown; the expression of *GDA* in KIJ265T cell line was on the border of detection limit). Statistical analysis was performed using one-way ANOVA with Dunnett’s Multiple Comparison Test, with exception of analysis of GATM and GOT1 for which t-test was used * *p* < 0.05, ** *p* < 0.01, ***p < 0.001. (**B)** The effects of miRNAs on the activity of luciferase reporter gene under control of cloned miRNA binding sites predicted in metabolic genes. Caki-2 cells were co-transfected with reporter plasmid bearing MRE (miRNA response element) for a given microRNA, and either microRNA mimic or non-targeting scrambled control oligonucleotides. The plots show results of three independent biological experiments. Statistical analysis was performed using Students t-test. (**C**) The effects of miR-155-5p on protein expressions of GATM in Caki-2 cells. Upper panel: Representative photographs of Western blots. Lower panel: Results of densitometric scanning of Western blots. The plot shows mean expression of GATM protein in three independent biological experiments performed in two-three replicates. * *p* < 0.05.

Basing on the assumption that the miRNAs the most strongly correlating with metabolic genes could have the greatest impact on cellular metabolism, we next evaluated the effects of the five miRNAs (miR-34a-5p, miR-106b-5p, miR-122-5p, miR-146a-5p, and miR-155-5p) on mRNA expression of metabolic genes (Figure 2A) that were predicted as possible targets for specific miRNAs (Table S1). For each miRNA, we analyzed only the expression of transcripts of which 3′UTRs possessed potential binding sites for this specific miRNA as indicated by the bioinformatic analysis. Transfections of miRNA mimics in two RCC-derived cell lines resulted in downregulation of GATM mRNA by miR-155-5p, GDA by miR-106b-5p and miR-146a-5p, and SUCLG2 by miR-146a-5p and miR-155-5p. In addition, miR-155-5p statistically significantly suppressed the expressions of GDA and PCCA in only one of the analyzed cell lines. In Caki-2 cells, the expression of ALDH5A1 was stimulated by miR-122-5p and miR-146a-5p, while the expression of ALDH6A1 was stimulated by miR-106b-5p and miR-122-5p. miR-342-3p concomitantly increased the expression of PCCA in both analyzed cell lines (Figure 2A). 

We subsequently evaluated the effects of miRNAs on protein expressions of metabolic genes. Firstly, we checked whether miRNAs could interact with sequences predicted as miRNA response elements (MREs) in target transcripts. To this end, the predicted binding sites were cloned into luciferase reporter system, which was co-transfected into RCC cells with miRNA mimics or non-targeting control oligonucleotides (Figure 2B). We found that miR-155-5p significantly suppressed luciferase activity under control of MREs cloned from *GATM* and *SUCLG2* sequences, while miR-106b-5p and miR-146a-5p decreased luciferase activity of two MREs cloned from *GDA*. Remarkably, no changes in luciferase activity were found when miRNA mimics were co-transfected with the reporter constructs with mutated MREs of *GATM*, *SUCLG2*, and *GDA* (Figure S2). We also observed miR-155-5p-mediated suppression of luciferase activity under control of MRE cloned from *PCCA*; however, this effect was not specific as indicated by experiments with mutated binding sequences (Figure S2). In accordance with the effect of miR-146a-5p on *ALDH5A1* mRNA (Figure 2A), luciferase activity was also increased when MRE cloned from *ALDH5A1* was treated with miR-146a-5p mimic (Figure 2B). However, miRNA mimics did not affect the activity of empty reporter vector (Figure S2). 

Finally, we analyzed the effects of miRNA mimics on the endogenous expression of proteins encoded by metabolic genes (Figure 2C). The expression of GATM was dramatically reduced by transfection with miR-155-5p in Caki-2 cells but not in KIJ265T cells (Figure S3). The expressions of ALDH5A1, ALDH6A1, and GDA proteins were not changed by transfection of the miRNA mimics (Western blots for these proteins are shown in Figure S3). Antibodies against PCCA gave non-specific signals and were, therefore, discarded from the analysis (Figure S3). 

### 2.2. Metabolic miRNAs Affect Proliferation of RCC Cells and Correlate with Poor Survival of RCC Patients

**Figure 3:**
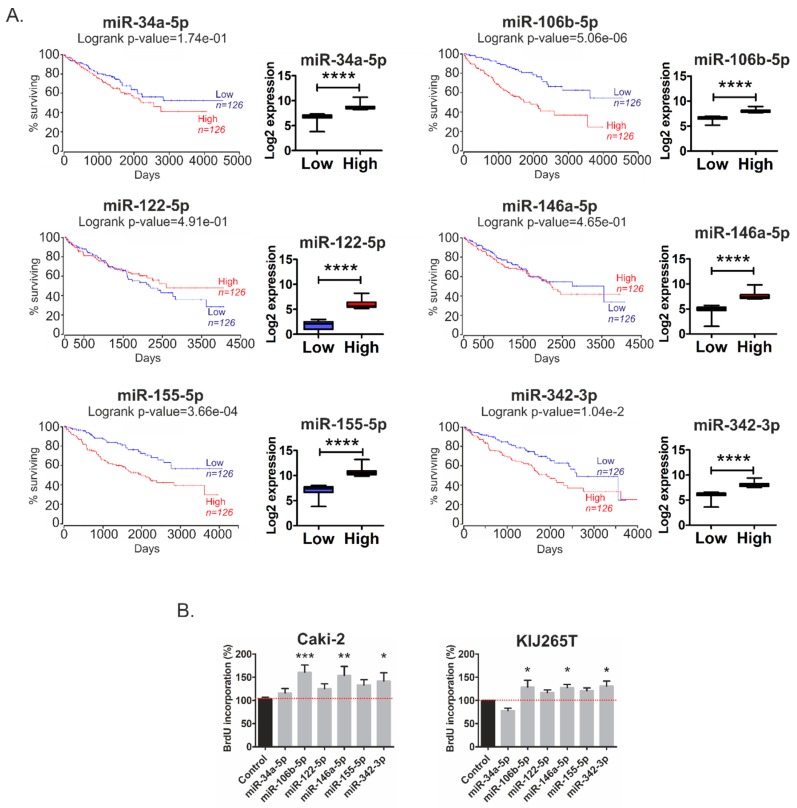
MicroRNAs effects on survival of RCC patients and proliferation of RCC cells. (**A**) Kaplan–Meier plots of RCC patients generated using OncoLnc tool and KIRC cohort of TCGA data. Patients were classified into Low and High expression groups basing on median miRNA expression data, which are shown on the graphs below the K-M plots. **** *p* < 0.0001; analysis was done using Mann–Whitney test. (**B**) The effects of microRNAs on proliferation of Caki-2 and KIJ265T cells. The plots show results of BrdU assay performed in three independent biological experiments. Statistical analysis was done using repeated measures ANOVA with Dunnett‘s Multiple Comparison post-test. * *p* < 0.05, ** *p* < 0.01, *** *p* < 0.001.

Given the above-described findings, we next looked for potential associations between altered expression of miRNAs targeting metabolic genes and survival of RCC patients. Analysis of publicly available TCGA data revealed that high expression of miR-106b-5p, miR-155-5p, and miR-342-3p correlated with poor survival of RCC patients (Figure 3A). There was no statistically significant correlation between the expressions of miR-34a-5p, miR-122-5p, and miR-146a-5p and survival of patients. We subsequently analyzed the effects of metabolic miRNAs on the proliferation of RCC cells. Transfection of miR-106b-5p, miR-146a-5p, and miR-342-3p concomitantly stimulated proliferation in both analyzed RCC cell lines. The proliferation of cells transfected with miR-122-5p and miR-155-5p was also increased, although without statistical significance (Figure 3B).

These results indicate that altered expression of metabolically-relevant miRNAs could possibly contribute to cancer progression and shorten the survival time of RCC patients.

### 2.3. MiR-146a-5p is a Global Regulator of Key Metabolic Pathways in RCC

**Figure 4:**
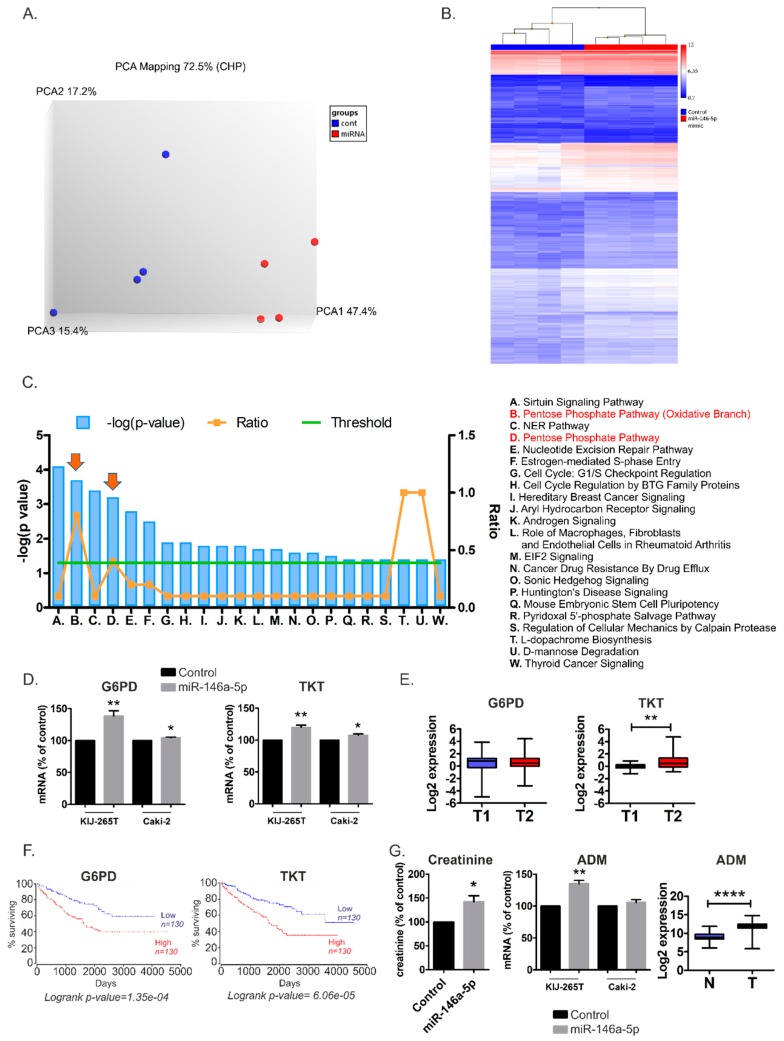
The effects of miR-146a-5p transfection in RCC cells. **(A)** Principal component analysis (PCA) of transcriptome data obtained from KIJ265T cell line transfected with miR-146a-5p mimic or non-targeting control oligonucleotide (Cont. (**B**) Hierarchical clustering based on differentially expressed genes generated using TAC 4.0. **(C)** Top pathways affected by miR-146a-5p transfection in RCC cells. The plot shows results of IPA Core Analysis performed on the genes affected by transfection of miR-146a-5p mimic (shown in Table S4). The overrepresented pathways are listed according to the –log (*p* value) (blue bars) (left y-axis). The threshold line (green) represents *p* value = 0.05. The ratio of the number of genes found in each pathway and the total number of genes in the pathway is shown in orange (right y-axis). PPP pathway is shown with arrows. **(D)** The expressions of genes involved in the pentose phosphate pathway (*G6PD*, *TKT*) are upregulated in RCC cells transfected with miR-146a-5p mimic. The effect of miR-146a-5p was analyzed in three independent biological experiments performed in triplicate. Statistical analysis was performed using t-test. * *p* < 0.05. ** *p* < 0.01. (**E**) The expression of *G6PD* and *TKT* in RCC tumors classified according to TNM system [1]. T1 (*n* = 30): tumors classified as Stages I and II (tumors limited to the kidney, with no signs of metastasis); T2 (*n* = 30): tumors classified as Stages III and IV (tumors which invade veins and neighboring structures as well as tumors with metastasis in lymph nodes or distant organs). Statistical analysis was performed using Mann–Whitney test. ** *p* < 0.01. (**F**) High expressions of *G6PD* and *TKT* correlate with poor survival of RCC patients. Kaplan–Meier plots of RCC patients were generated using OncoLnc tool and KIRC cohort of TCGA data. Patients were classified into Low and High expression groups basing on median gene expression data. (**G**) miR-146a-5p transfection increases creatinine levels in RCC cells. Left panel: The plot shows results of GC-MS analysis of RCC cells transfected with miR-146a-5p mimic or non-targeting control oligonucleotide. Middle panel: The expression of adrenomedullin (*ADM*) is increased in KIJ265T RCC cells transfected with miR-146a-5p mimic. Right panel: The expression of *ADM* is increased in RCC tumors (T, *n* = 250) when compared with control kidney samples (C, *n* = 72). The analysis was performed using publicly available transcriptomic data of TCGA consortium (KIRC cohort). Statistical analysis was performed using t-test. * *p* < 0.05. ** *p* < 0.01. **** *p* < 0.0001.

Next, we asked whether one specific miRNA could globally affect RCC metabolism. To answer this question, we implemented microarray analysis of RCC cells transfected with miR-146a-5p mimic or non-targeting control oligonucleotide. miR-146a-5p was selected for two reasons: firstly, it significantly stimulated proliferation of RCC cells (Figure 3B), indicating genuine reprogramming of cells functioning. Secondly, miR-146a-5p is the first miRNA for which functional interaction with TCA cycle was recently provided in vivo [9] and alterations of TCA cycle are a characteristic feature of RCC tumors [3]. The principal component analysis (PCA) and hierarchical clustering of RCC cells transfected with a miR-146a-5p mimic or non-targeting control oligonucleotide proved robustness of the obtained datasets and clear distinctiveness of compared groups (KIJ265T cell line transfected with miR-146a-5p mimic and transfected with non-targeting control oligonucleotide) (Figure 4A,B). 

Transcriptome Analysis Console (TAC) software evaluation revealed the altered expression of 955 genes, including 810 up-regulated in 145-down-regulated transcripts (Table S4). TAC analysis revealed that miR-146a-5p affected the expressions of genes involved in the TCA cycle; the OXPHOS system in mitochondria; the pentose phosphate pathway (PPP); metabolism of amino acids, nucleotides, and glutathione; adipogenesis; fatty acids beta-oxidation; trans-sulfuration; and one-carbon metabolism (Table 2). 

Both TAC (Table S5) and Ingenuity Pathway Analysis (Figure 4C) revealed that the pentose phosphate pathway was among the most altered metabolic pathways in miR-146a-5p transfected cells. qPCR validation confirmed that miR-146a-5p transfection induced expression of *G6PD* and *TKT*, two key genes encoding PPP enzymes (Figure 4D). The expression of *TKT* was higher in more advanced RCC tumors than in less advanced lesions. For *G6PD*, no statistically significant expression changes were observed (Figure 4E). Remarkably, high *TKT* and *G6PD* expressions in tumors significantly correlated with poor survival rates of RCC patients (Figure 4F), which may partially reflect the pro-proliferative effects of their stimulator, miR-146-5p. To analyze the impact of miR-146a-5p on metabolic profile of RCC cells, we performed GC-MS analysis of RCC cells transfected with the miR-146a-5p mimic. This analysis revealed increased levels of creatinine in KIJ265T cells transfected with miR-146a-5p when compared with those transfected with a non-targeting control oligonucleotide (Figure 4G). Interestingly, microarray analysis (Table S4) and qPCR validation in KIJ265T cells (Figure 4G) indicated that miR-146a-5p transfection caused upregulation of *ADM*, a gene encoding adrenomedullin which contributes to creatinine clearance [10,11,12], suggesting cellular response to increased creatinine levels. In Caki-2 cells, the expression of *ADM* was not statistically significantly changed following miR-146a-5p (Figure 4G). In accordance, GC-MS analysis revealed that creatinine levels were not statistically significantly changed in Caki-2 cells transfected with miR-146a-5p (not shown). 

### 2.4. Metabolically-Relevant miRNAs Regulate the Expression of NFAT5

**Figure 5:**
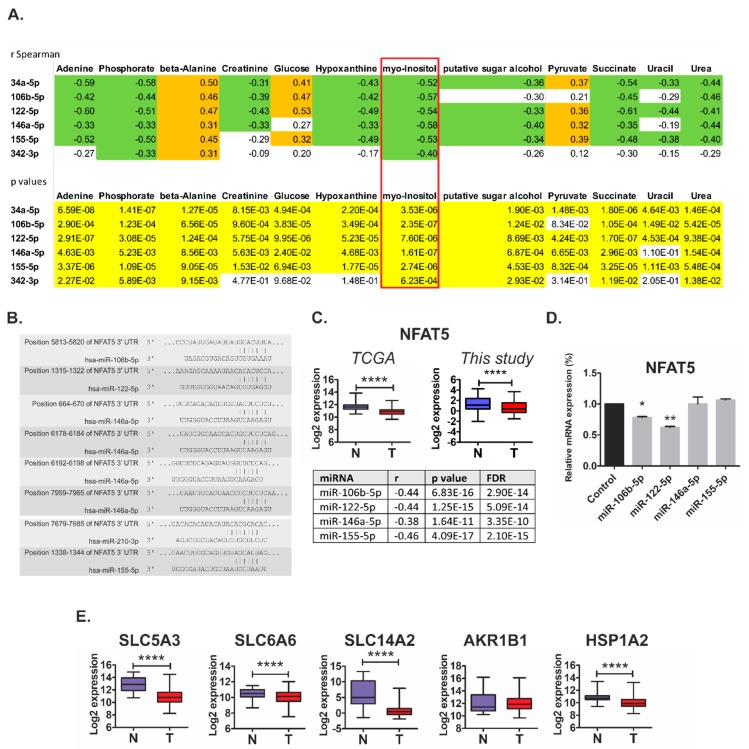
Osmoregulatory NFAT5 as a target of metabolically-relevant miRNAs in renal cancer. **(A)** Correlations between the expressions of microRNAs and metabolite levels in tissue samples from 70 control and RCC samples. Upper panel: Correlation coefficients. Orange: r Spearman > 0.3; green: r Spearman < −0.3. Lower panel: *p* values; yellow: *p* < 0.05. Full data of correlation analysis is given in Table S6. (**B**) The potential binding sites of miRNAs in *NFAT5* 3′UTR, predicted by TargetScan. **(C)** Upper panel: The expression of *NFAT5* is decreased in RCC tumors (TCGA cohort: T, *n* = 250; this study cohort: T, *n* = 60) when compared with control kidney samples (TCGA cohort: C, *n* = 72; this study cohort: C, *n* = 60). Statistical analysis was performed using t-test. **** < 0.0001. Lower panel: Negative correlations between the expressions of NFAT5 and the predicted microRNAs. Correlation analysis was performed using StarBase v2.0. on KIRC cohort of RCC patients (*n* = 300). For miR-210-3p, no data were available. **(D)** The expression of *NFAT5* mRNA is suppressed by miR-106b-5p and miR-122-5p in RCC cell line. Caki-2 cells were transfected with mimics of the respective microRNAs or non-targeting scrambled oligonucleotides. The plots show the results of three independent biological experiments. Statistical analysis was performed using repeated measures ANOVA with Dunnett’s Multiple Comparison post-test. * *p* < 0.05, ** *p* < 0.01. (**E**) The expression of *NFAT5* target genes is decreased in RCC tumors (T, *n* = 250) when compared with control kidney samples (N, *n* = 72). The analysis was performed using publicly available transcriptomic data of TCGA consortium (KIRC cohort). Statistical analysis was performed using Students *t*-test. **** < 0.0001.

The fact that miR-146a-5p influenced the level of only one metabolite (creatinine) suggested that the combined action of several microRNAs may be required for reprogramming of cancer cell metabolism. In the search for such possible cooperative effects of miRNAs on RCC metabolism, we analyzed correlations between the expression of the 22 initially identified miRNAs and the levels of 54 metabolites in RCC tissue samples (Table S6). Strikingly, we found that miR-34a-5p, miR-106b-5, miR-122-5p, miR-146a-5p, and miR-155-5p were among the miRNAs with the highest number of correlating metabolites (Table S6). Furthermore, we found that expression of these microRNAs commonly correlated with similar metabolites. In particular, we found strong negative correlations (r Spearman ≤ −0.4, *p* < 0.001) between the expressions of all five microRNAs and the levels of *myo*-inositol (Figure 5A).

On the basis of these observations, we searched for the possible target genes that could mediate cooperative actions of miRNAs associated with metabolic changes in RCC tumors. To this end, we next selected miRNAs whose expression was most strongly negatively (r Spearman < −0.5) correlated with *myo*-inositol levels (Table S6), and searched for their potential target genes using the miRsystem platform that incorporates seven independent prediction algorithms [13]. Remarkably, we found *NFAT5* as the top gene, predicted to be commonly co-regulated by five out of seven analyzed microRNA: miR-106b-5p, miR-122-5p, miR-146a-5p, miR-155-5p, and miR-210-3p (Figure 5B and Table S7). Furthermore, we found that *NFAT5* expression in renal tumors is decreased (Figure 5C), which fits the profile of increased expression of the predicted targeting microRNAs. We also found strong negative correlations between the expression of *NFAT5* and the four predicted miRNAs in RCC tumor samples (Figure 5C) and other types of cancer (File S2). These results suggest that *NFAT5* could indeed be a common target of the miRNAs that affect *myo*-inositol levels in tumor tissues. Transfection with mimics of miR-106b-5p and miR-122-5p suppressed the expression of *NFAT5* in RCC cell line (Figure 5D). NFAT5 is an osmoprotective transcription factor that controls expression of genes that counteract signals inducing cell shrinkage during osmotic stress. The key NFAT5 targets are SLC5A3 (a *myo*-inositol transporter), SLC6A6 (a beta-alanine transporter), AKR1B1 (aldose reductase; catalyzes reduction of glucose to sorbitol), SLC14A2 (a urea transporter), and HSPA1B (a chaperone protecting cells against apoptosis induced by urea [14]. The expressions of most of these genes were decreased in renal tumors (Figure 5E). The only exception was *AKR1B1* for which expression was unaltered. Taken together, these results indicate that miRNA-mediated changes in NFAT5 expression could contribute to changed levels of osmolytes (e.g., *myo*-inositol) via altered expression of the proteins responsible for their transport in RCC cells. 

### 2.5. MiR-34a-5p, miR-106b-5p, miR-146a-5p and miR-155-5p Are PanCancer MetabomiRs

**Figure 6:**
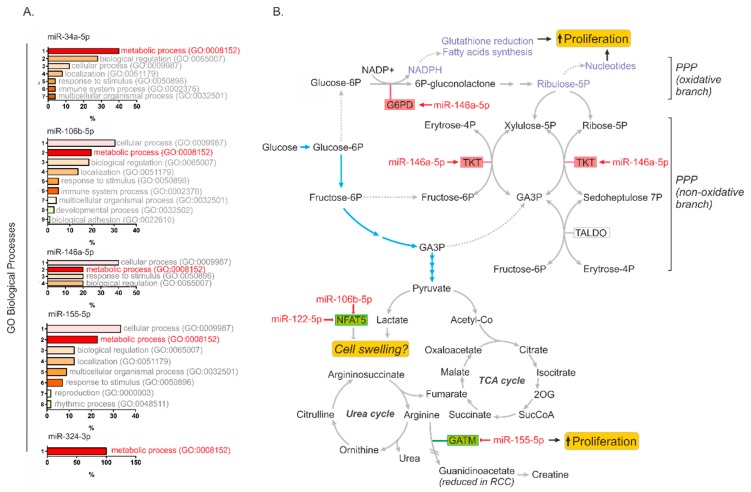
microRNA-mediated regulation of cancer metabolism. (**A**) Functional annotation of genes predicted as targets of microRNAs identified in our study in PanCancer analysis encompassing 14 cancer types and >6000 patients. Only genes for which expression correlated with a given microRNA in at least 10 cancer types were selected for the analysis. The list of genes is provided in Table S7. The plots show results of PANTHER Functional classification analysis according to GO Biological processes annotated to the predicted genes. (**B**) The model showing microRNAs affecting key metabolic pathways in RCC cells: miR-146a-5p upregulates key PPP genes (G6PD and TKT), thereby contributing to enhanced cancer cell proliferation; miR-155-5p suppresses the expressions of gene involved arginine metabolism (GATM); and miR-106b-5p and miR-122-5p may possibly counteract cell swelling induced by enhanced lactate production, by suppressing the expression of NFAT5, which governs the activity of genes encoding proteins transporting osmolytes (e.g., *myo*-inositol). Abbreviations: GA3P, glyceraldehyde-3-phosphate; 2OG, 2-oxoglutarate. Glycolysis is shown with blue arrows.

On the basis of the collected data presented above, we hypothesized that miRNAs identified in our study could be involved in global regulation of cancer metabolism. To this end, we searched for possible correlations between the expression of miR-34a-5p, miR-122-5p, miR-146a-5p, miR-155-5p, and miR-342-3p and their predicted target genes in the transcriptomes of 14 types of cancers in more than 6000 samples (Tables S8 and S9). Next, we selected miRNA targets of which expressions correlated in at least ten cancer types and performed PANTHER Functional Classification Test to find biological processes annotated to the analyzed genes (Figure 6A). Strikingly, “metabolic process” emerged at the top of annotated processes for most miRNAs targets. The only exception was miR-122-5p for which no gene targets were found which correlated in at least 10 cancer types. These results indicated that miR-34a-5p, miR-106b-5p, miR-146a-5p, and miR-155-5p could represent PanCancer metabo-miRs, involved in global regulation of cellular metabolism in cancer cells. 

## 3. Discussion

In this paper, we present a group of microRNAs that regulate genes involved in key metabolic pathways and contribute to enhanced proliferation of renal cancer cells. We show that microRNAs can affect the RCC metabolome both directly (e.g., miR-155-5p targeting GATM) and indirectly, by cooperative regulation of the expression of NFAT5, a transcription factor governing the expression of transporters that control osmolality. We also show that miR-146a-5p globally affects the expression of genes involved in key metabolic pathways in RCC such as those associated with the PPP. Finally, the results of PanCancer analysis indicate that miR-34a-5p, miR-106b-5p, miR-146a-5p, miR-155-5p, and miR-342-3p may be involved in global regulation of metabolism in cancers of various origins.

Upregulation of the pentose phosphate pathway (PPP) is one of the key features of the dysregulated metabolism of RCC cells [15]. It enables efficient production of NADPH, utilized as a reducing agent contributing to redox homeostasis of cancer cells, and ribose-5-phosphate, required to support the high rates of nucleotide synthesis during intensive malignant proliferation [16] (Figure 6B). G6PD is a rate-limiting enzyme of the PPP and its inhibition attenuates survival of RCC cells [17]. We found that miR-146a-5p stimulated the expression of *G6PD* and *TKT*, the genes encoding two key enzymes of the oxidative and non-oxidative PPP branches, respectively. Interestingly, it was shown that transketolase (TKT) activity correlates with creatinine levels in uremic patients [18], which may possibly partially explain the observation that creatinine levels increased following miR-146a-5p transfection. Remarkably, other reports also demonstrated that creatinine concentrations can be affected by miRNAs [19,20]. Most metabolic genes affected by miR-146a-5p transfection exhibited upregulated gene expression (Table 2). This suggests that miR-146a-5p-mediated transcriptomic effects were not direct. Possible mediatory mechanisms may include activation of transcription regulators (e.g., E2F4 and NCOR2), mRNA processing factors or the suppression of inhibitory microRNAs (e.g., miR-29a) (Table S3). However, the exact mechanisms mediating miR-146a-5p-induced upregulation of gene expression remains to be delineated in the future. 

*GATM* (*AGAT*) encodes glycine amidinotransferase, a mitochondrial enzyme that catalyzes the transfer of a guanido group from L-arginine to glycine, resulting in guanidinoacetic acid, which is a substrate for creatine synthesis. Suppressed expression of GATM in RCC tumors [4] is in line with recent findings of decreased excretion of guanidinoacetate (GAA) in RCC patients [21]. Interestingly, several studies have demonstrated that creatine inhibits growth of tumor cells both in vitro and in vivo [22,23,24]. The exact mechanism by which creatine attenuates cancer growth is unknown; however, possible mechanisms include inhibition of glycolysis or generation of acidosis [25]. It may thus be hypothesized that miR-155-5p-mediated downregulation of GATM in RCC cells may lead to a reduction of intracellular creatine pool, thereby preventing its anticancer activities. A possible tumor-suppressive role of GATM is supported by the fact that its low expression correlates with poor survival rates of RCC patients (Figure 1C). By contrast, high expression of miR-155-5p, which downregulates GATM, correlates with poor prognosis for RCC patients (Figure 3). We did not see the suppression of GATM protein by miR-155-5p in KIJ265T cells (Figure S3). This observation is in agreement with previous studies that showed that miR-155 regulates gene expression in a cell-type specific manner [26,27]. 

Intensive proliferation and metabolic activation of cells lead to osmotic stress which results from enhanced consumption of metabolites that function as intracellular osmolytes, such as *myo*-inositol or amino acids [28]. Our results suggest that enhanced expression of microRNAs, in particular miR-106b-5p and miR-122-5p, may contribute to osmotic stress by inhibiting the expression of *NFAT5*, a transcription factor that regulates gene expression in response to osmotic challenge [14,29]. Mammalian cells exposed to hypertonic environment respond by releasing water, and activating NFAT5, which in turn leads to accumulation of intracellular organic osmolytes, i.e., betaine, taurine, and *myo*-inositol [29]. NFAT5 is also involved in the regulation of cell survival, migration, proliferation and angiogenesis [29]. Furthermore, the possible role of NFAT5 in cancer is supported by the fact that genes encoding proteins involved in the transport of osmoregulators are markers of the cancer phenotype [30]. NFAT5 plays different functions during cancer development and progression. In melanoma cells, it stimulates invasion [31], while in thymoma it promotes T cells proliferation and activation [32]. In hepatocellular carcinoma, NFAT5 functions as a tumor suppressor and promotes apoptosis with concomitant inhibition of cell cycle progression [33]. *NFAT5* expression is regulated by multiple microRNAs, including miR-211 in melanoma [31]; miR-641 in glioma [34]; miR-1b, miR-106a, and miR-363-3p in differentiating Th17 cells [35]; miR-22 in colon cancer [36]; and miR-568 during Treg cells activation [37]. Furthermore, NFAT5 is a target of a group of osmoresponsive miRNAs that regulate its expression during osmoadaptation in mice [38]. During cell growth, NFAT5 regulates cell volume [28], which is influenced by constant changes in extracellular and intracellular osmolality. Changes in cell volume may affect concentrations of key signaling molecules, thereby influencing proliferation, migration, and cell death [39]. Persistent changes in cell volume can lead to necrotic volume increase (NVI) and finally to cell death [40]. The key molecular features of RCC pathology are metabolic reprogramming associated with enhanced lactate production and activation of hypoxia-induced signaling pathways [3]. Remarkably, both intracellular lactate accumulation and hypoxia can stimulate cell swelling [40,41,42]. Depletion of intracellular *myo*-inositol is a well-known mechanism that counteracts cell swelling [43,44]. Reduction of *myo*-inositol levels in the kidney may result from its reduced uptake by transporting proteins such as SLC5A3 [45]. It may thus be hypothesized that microRNA-induced changes in expression of NFAT5 and the resulting reduced expression of *myo*-inositol transporter may represent a mechanism which protects RCC cells against cell swelling-induced death. 

Our study took advantage of the publicly available data of the PanCancer project [46]. This initiative of the TCGA consortium aims to analyze similarities and differences between different tumor types and tissue sites of origin. Since its launch in 2012, the PanCancer project has resulted in plethora of novel findings, including the importance of cell-of-origin in tumor pathology [47], development of clinical outcome endpoints recommended for 33 cancer types [48], new clustering of tumor types that may be implemented in future clinical trials [49], information on potential targets for new combinations therapies [50], and the collection of digitalized histopathological sections from more than 11,000 patients [51] that are already used for creation of new bioinformatic diagnostic tools. Since it was recently revealed that PanCancer metabolic profiling allows for prediction of responses to therapy [52], we hope that the results of our study will bring the basis for future research focused on finding better therapeutic options of RCC patients. 

## 4. Materials and Methods 

### 4.1. Tissue Samples

Ninety-five RCC tumor tissue samples and 95 matched-paired non-tumorous control kidney samples (190 tissue samples in total) were from the local Tissue Bank stored at −80 ^°^C at the Department of Biochemistry and Molecular Biology, Centre of Postgraduate Medical Education. Collection of tissue samples was performed under approval of the Bioethical Committee of Centre of Postgraduate Medical Education (No. 18/PB/2012 and No. 75/PB-A/2014), with written informed consent obtained from patients. 

### 4.2. Cell Lines

Caki-2 cell line was purchased from ATCC (Manassas, VA, USA). KIJ-265T cell line was a kind gift of Dr. John A. Copland and Mayo Foundation for Medical Education and Research (Rochester, MN, USA). Both cell lines were cultured in accordance with providers’ instructions.

### 4.3. Transfections

The cells were seeded on 12-well, 6-well or 60-mm plates in complete medium and cultured for 24 h. Transfections were performed as described previously [6] using miRCURY LNA microRNA mimics/inhibitors or control oligonucleotides provided in Table S10. 

### 4.4. Isolations of RNA and Proteins, Reverse Transcription

They were performed as previously described [6]. qPCR array analysis using Custom Panel (Roche Diagnostics, Mannheim, Germany) and Pick&Mix microRNA PCR Panels (Exiqon, Vedbaek, Denmark) were performed as previously [4,6]. Primers and probes for qPCR reactions are given in Tables S11 and S12. The expression of microRNAs in tissue samples was normalized against miR-103a-3p, for which stable expression was confirmed (Figure S4).

### 4.5. Cloning of miRNA Targets Sites and Luciferase Assays

They were performed using pmiRGLO reporter vector as provided in our previous study [6]. Sequences of oligonucleotides used for cloning of miRNA target sites are provided in Table S13.

### 4.6. Western Blots

WB were performed as in a previous study [53]. Details on antibodies and dilutions are given in Table S14. 

### 4.7. Analysis of Proliferation

Proliferation of RCC cells was analyzed using BrdU assay (Roche Diagnostics, Mannheim, Germany) and an earlier described procedure [53]. 

### 4.8. Transcriptomic Analysis

For transcriptomic analysis, we used RNA isolated from four independent wells of a 12-well plate, transfected with miR-146a-5p mimic, and four independent wells of a 12-well plate, transfected with non-targeting control oligonucleotide. Microarray analysis was performed using Affymetrix Gene Atlas System according to the manufacturer’s instructions. Briefly, 150 ng of total RNA that passed initial quality control screen (2100 Bioanalyzer, Agilent, Santa Clara, CA, USA) was used for target preparation using the GeneChip™ WT PLUS Reagent Kit (ThermoFisher Scientific, Waltham, MA, USA). Prepared samples were hybridized to the Affymetrix™ HuGene 2.1 ST Array Strips (Affymetrix, Santa Clara, CA, USA). Arrays after washing and staining, were scanned in Gene Atlas Imaging Station (Affymetrix) with .CEL files as data output. Data analysis was performed using Transcriptome Analysis Console (TAC) Software 4.0 (ThermoFisher) and Ingenuity Pathway Analysis Software (IPA, QIAGEN Bioinformatics, Hilden, Germany). After importing Human Gene 2.1 ST .CEL files into TAC 4.0, the array data were normalized by the RMA method. The probe summarization and the microarray quality control were done with TAC 4.0 according to the manufacturer’s instructions. In the next step, TAC 4.0 one-way ANOVA was utilized to determine differentially expressed genes (DEGs) between treatment and control: KIJ265T cell line transfected with miR-146a-5p mimic or transfected with non-targeting control oligonucleotide, respectively. To minimize the variability originating from different sample preparation dates in a comparison analysis, TAC 4.0 batch effect was applied. The criteria for selecting DEGs were fold change ≤ −1.5 or fold change ≥ 1.5 and *p* value ≤ 0.05. Further bioinformatic analyses were performed with TAC 4.0 and Ingenuity Pathway Analysis Software (IPA, Qiagen Bioinformatics). The results of microarray analysis were validated using qPCR. To validate microarrays experiments, transfections with miR-146a-5p and non-targeting control oligonucleotides were repeated three times on independent days. Each day, transfections were performed using three wells of a 12-well plate for miR-146a-5p mimic and three wells of a 12-well plate for control oligonucleotide. 

### 4.9. Metabolomic Analysis

Cells were cultured in medium without phenol red. Then, 72 h after transfection, cells were washed five times with PBS and metabolites were extracted with 1 mL of 1:3 methanol: MTBE extraction buffer containing internal standards (500 ng of 1,2-diheptadecanoyl-sn-glycero-3-phosphocholine (Avanti Polar Lipids, 850360P) and 500 ng of ^13^C sorbitol. Metabolite profiling was performed using a gas chromatography mass spectrometer (GC-MS) as earlier described [4]. To measure cellular proteins, the cell residues were resuspended in 0.1 M NaOH containing 0.125% Triton X-100 [54]. 

### 4.10. Bioinformatics Analysis

Prediction of microRNAs targeting genes involved in metabolic pathways was performed using miRSystem (http://mirsystem.cgm.ntu.edu.tw/microrna.org [13]), TargetScan [55] and literature search according to the criteria described in File S1. miRsystem parameters were defined to include validated genes greater than or equal to 3 and O/E ratio greater than or equal to 2. Survival analysis was performed using OncoLnc tool (f http://www.oncolnc.org) [56] and SurvExpress (http://bioinformatica.mty.itesm.mx:8080/Biomatec/SurvivaX.jsp) [57] using KIRC cohorts of TCGA transcriptomic data. The expressions of ADM, NFAT5 and its target genes were analyzed using FireBrowse RESTful API visual interface (http://firebrowse.org/api/api-docs, api version: 1.1.38) on KIRC cohort data. Analysis of correlations between NFAT5 and miRNAs was performed using StarBase v2.0. on PanCancer data involving 14 types of cancer [58]. 

### 4.11. Statistical Analysis

Data distribution was analyzed using Shapiro–Wilk test. Statistical significance of two groups of data was analyzed using t-test, Wilcoxon matched-pairs signed rank test or Mann–Whitney test. Correlations were analyzed using Spearman r or Pearson r, depending on data distribution. Analysis of more than two groups of data was performed using one-way ANOVA with Dunnett’s Multiple Comparison Test. *p* < 0.05 was considered statistically significant. Statistical analyses were done using GraphPad Prism 5.00 for Windows.

## 5. Conclusions

We found that increased expression of microRNAs in renal cancer contributes to disturbed expression of key genes involved in the regulation of RCC metabolome. The correlations between microRNAs expression and the profiles of RCC metabolites suggest that changes in expression of small non-coding microRNAs may contribute to the metabolic reprogramming and osmoregulation in renal tumors. In particular, miR-146a-5p and miR-155-5p emerge as a key “metabomiRs” that target genes of crucial metabolic pathways (PPP, TCA cycle, arginine metabolism) in RCC, while enhanced expression of miR-106b-5p may contribute to dysregulation of osmotic control in renal cancer cells. The fact that altered expressions of miR-106b-5p, miR-155-5p, and miR-342-3p correlate with poor survival of RCC patients strengthens their significance as oncogenic microRNAs in RCC. Finally, the results of our study indicate that miR-34a-5p, miR-106b-5p, miR-146a-5p, miR-155-5p, and miR-342-3p are PanCancer metabomiRs that may be involved in global regulation of cancer metabolism.

## Figures and Tables

**Table 1:** The expressions of genes involved in the regulation of cell metabolism and their predicted regulatory miRNAs are altered in RCC tumor tissues.

**A. Expression of Metabolic Genes in RCC**		
**Gene**	**FC**	***p* Value**
Increased expression in tumors		
*1. ADA*	+5.77	<0.0001
*2. IL4I1*	+4.20	<0.0001
*3. HK3*	+3.96	<0.0001
*4. PYCR1*	+1.56	<0.0001
Decreased expression in tumors		
*5. PAH*	−70.47	<0.0001
*6. ALDH6A1*	−21.41	<0.0001
*7. CMKT2*	−18.36	<0.0001
*8. ALDH4A1*	−14.72	<0.0001
*9. GATM*	−12.99	<0.0001
*10. DPYS*	−10.83	<0.0001
*11. G6PC*	−10.83	<0.0001
*12. PCCA*	−6.87	<0.0001
*13. GPT*	−6.62	<0.0001
*14. GDA*	−6.37	<0.0001
*15. ALDH5A1*	−5.54	<0.0001
*16. SUCLG2*	−5.35	<0.0001
*17. ARG2*	−4.45	<0.0001
*18. GOT1*	−3.68	<0.0001
*19. PHOSPHO1*	−1.35	=0.0215
**B. Expression of miRNAs Predicted to Regulate Metabolic Genes in RCC**		
**MicroRNA**	**FC**	***p* Value**
Increased expression in tumors		
1. miR-122-5p	+107.7	<0.0001
2. miR-210-3p	+10.2	<0.0001
3. miR-155-5p	+8.3	<0.0001
4. miR-34a-5p	+3.1	<0.0001
5. miR-146a-5p	+2.1	<0.0001
6. miR-106b-5p	+2.1	<0.0001
7. miR-342-3p	+1.9	<0.0001
8. miR-454-3p	+1.6	<0.0001
9. miR-28-5p	+1.5	<0.0001
10. miR-126-3p	+1.5	<0.0001
11. miR-340-5p	+1.5	<0.0001
12. miR-20-5p	+1.4	<0.0001
Decreased expression in tumors		
13. miR-129-1-3p	−17.0	<0.0001
14. miR-129-2-3p	−6.6	<0.0001
15. miR-200b-3p	−4.3	<0.0001
16. miR-370-3p	−2.6	<0.0001
17. miR-20b-5p	−2.4	<0.0001
18. miR-133a-3p	−2.2	0.0262
19. miR-154-5p	−2.1	<0.0001
20. miR-135b-5p	−2.0	0.0003
21. miR-27b-3p	−1.6	<0.0001
22. miR-543	−1.5	0.0337

(**A**) The expression of metabolic genes. (**B**) The expressions of microRNAs predicted to target metabolic genes. FC: fold change (the ratio between median expressions in tumor and control tissue samples); threshold = 1.3. *n* = 60 (RCC tumor samples), *n* = 60 (paired-matched control samples). Statistical analysis was performed using Wilcoxon matched-pairs signed rank test. MicroRNAs selected for functional analysis are bolded.

**Table 2:** miR-146a-5p affects expression of genes involved in key metabolic pathways. The table shows selected DEGs in RCC cells transfected with miR-146a-5p mimic, compared to cells transfected with non-targeting control oligonucleotide with functions in different metabolic pathways identified by biological pathway analysis with WikiPathways included in TAC 4.0.

Symbol	Entrez Gene Description	Metabolic Pathway	Fold Change	*p*-Value
*ACO2*	aconitase 2	TCA cycle, Amino acid metabolism, Metabolic reprogramming in colon cancer	1.53	3.40 × 10^−3^
*AHCY*	Adenosylhomocysteinase	Trans-sulfuration pathway; Trans-sulfuration and one carbon metabolism	1.76	6.00 × 10^−4^
*ALDH1A1*	aldehyde dehydrogenase 1 family member A1	Tryptophan metabolism	2.2	5.00 × 10^−4^
*CANT1*	calcium activated nucleotidase 1	Pyrimidine metabolism	1.53	1.14 × 10^−2^
*CBS/CBSL*	cystathionine-beta-synthase	Amino acid metabolism; Trans-sulfuration pathway; Trans-sulfuration and one carbon metabolism; One carbon metabolism and related pathways	1.57	2.00 × 10^−4^
*CEBPD*	CCAAT enhancer binding protein delta	Adipogenesis	1.58	4.50 × 10^−3^
*CHDH*	choline dehydrogenase	One carbon metabolism and related pathways	1.63	4.50 × 10^−3^
*CKB*	creatine kinase B	Trans-sulfuration; Urea cycle and metabolism of amino groups	1.58	5.13 × 10^−2^
*CPT2*	carnitine palmitoyltransferase 2	Fatty Acids Beta Oxidation	1.61	2.20 × 10^−3^
*DHODH*	dihydroorotate dehydrogenase (quinone)	Pyrimidine metabolism	1.88	1.00 × 10^−4^
*DNMT3B*	DNA methyltransferase 3 beta	Trans-sulfuration; Trans-sulfuration and one carbon metabolism; One carbon metabolism and related pathways	1.5	6.20 × 10^−3^
*E2F1*	E2F transcription factor 1	Adipogenesis	1.82	9.00 × 10^−4^
*E2F4*	E2F transcription factor 4	Adipogenesis	2.01	8.00 × 10^−4^
*ECHS1*	enoyl-CoA hydratase, short chain 1	Fatty Acid Biosynthesis; Fatty Acid Beta oxidation; Tryptophan metabolism	1.55	1.29 × 10^−2^
*ECSIT*	ECSIT signalling integrator	Mitochondrial complex I assembly model OXPHOS system	1.61	3.60 × 10^−3^
*ENTPD4*	ectonucleoside triphosphate diphosphohydrolase 4	Pyrimidine metabolism	1.58	3.42 × 10^−1^
*ESRRA*	estrogen related receptor alpha	Energy metabolism	1.69	1.00 × 10^−4^
*G6PD*	glucose-6-phosphate dehydrogenase	Pentose Phosphate Pathway; Metabolic reprogramming in colon cancer; Glutathione metabolism	1.64	6.00 × 10^−4^
*GK*	glycerol kinase	Fatty Acids Beta Oxidation	-1.75	4.30 × 10^−3^
*GPX4*	glutathione peroxidase 4	One carbon metabolism and related pathways; Glutathion metabolism	1.82	3.40 × 10^−3^
*H6PD*	hexose-6-phosphate dehydrogenase/glucose 1-dehydrogenase	Pentose Phosphate Pathway	1.72	4.40 × 10^−3^
*IDH2*	isocitrate dehydrogenase (NADP (+)) 2, mitochondrial	TCA cycle; Metabolic reprogramming in colon cancer	1.91	9.25 × 10^−5^
*LMNA*	lamin A/C	Adipogenesis	1.77	8.90 × 10^−3^
*LPIN3*	lipin 3	Adipogenesis	2.13	2.30 × 10^−3^
*MEF2D*	*myo*cyte enhancer factor 2D	Adipogenesis; Energy metabolism	1.7	1.83 × 10^−2^
*MYBBP1A*	MYB binding protein 1a	Energy metabolism	1.77	6.00 × 10^−4^
*NDUFAF8*	NADH:ubiquinone oxidoreductase complex assembly factor 8	Electron Transport Chain (OXPHOS system in mitochondria)	1.55	8.00 × 10^−4^
*NDUFB7*	NADH:ubiquinone oxidoreductase subunit B7	Electron Transport Chain (OXPHOS system in mitochondria); Mitochondrial complex I assembly model OXPHOS system	1.64	3.67 × 10^−2^
*NDUFS3*	NADH:ubiquinone oxidoreductase core subunit S3	Electron Transport Chain (OXPHOS system in mitochondria); Mitochondrial complex I assembly model OXPHOS system	1.52	9.00 × 10^−4^
*PGAM5*	PGAM family member 5, mitochondrial serine/threonine protein phosphatase	Metabolic reprogramming in colon cancer	1.52	1.20 × 10^−2^
*PGLS*	6-phosphogluconolactonase	Pentose Phosphate Pathway	1.53	6.40 × 10^−3^
*PYCR2*	pyrroline-5-carboxylate reductase 2	Metabolic reprogramming in colon cancer	1.5	6.00 × 10^−3^
*RAPGEF3*	Rap guanine nucleotide exchange factor 3	Integration of energy metabolism	1.58	4.00 × 10^−4^
*SDHA*	succinate dehydrogenase complex flavoprotein subunit A	Amino acid metablism; TCA cycle	1.52	2.60 × 10^−2^
*SEMA6B*	semaphorin 6B	TCA cycle	1.5	1.60 × 10^−3^
*SHPK*	sedoheptulokinase	Pentose Phosphate Pathway	1.63	4.00 × 10^−4^
*SOCS3*	suppressor of cytokine signaling 3	Adipogenesis	1.53	1.28 × 10^−2^
*STK11*	serine/threonine kinase 11	Integration of energy metabolism	1.69	6.00 × 10^−4^
*TKT*	Transketolase	Pentose Phosphate Pathway; Metabolic reprogramming in colon cancer	1.56	2.00 × 10^−3^

## Supplementary Materials

The following are available online at https://www.mdpi.com/2072-6694/11/12/1825/s1: Table S1: Selection of 20 genes for validation analysis performed in this study. Table S2: Preliminary analysis of expression of 90 microRNAs predicted to target genes involved in metabolic pathways in RCC tumor tissues. Table S3: Analysis of correlations between the expressions of miRNAs and their predicted target genes. Table S4: The results of microarray analysis of KIJ265T cells transfected with miR-146a-5p mimic or non-targeting control oligonucleotide. (XLSX). Table S5: Pathways enriched in KIJ265T cells transfected with miR-146a-5p mimic compared to cells transfected with control oligonucleotide. Table S6: Analysis of correlations between the expressions of miRNAs and levels of metabolites in RCC tissue samples. Table S7: The search for genes commonly targeted by miRNAs for which expressions correlated with myo-inositol levels in RCC tissue samples. Table S8: PanCancer analysis of correlations between the expression of microRNAs and their target genes. Table S9: Analysis of correlations between the expressions of microRNAs and their predicted target genes. Table S10: microRNA mimics, inhibitors, and control oligonucleotides used for transfections. Table S11: qPCR primers. (DOCX). Table S12: microRNA LNA primers used in the study. Table S13: Oligonucleotides used for cloning of miRNA target sites. (DOCX). Table S14: Antibodies used in Western blots. (DOCX). Figure S1: The expression profiles of metabolically relevant genes predicted as targets for microRNAs in two groups of patients stratified into “Low” (*n* = 130) and “High” (*n* = 130) expression groups. Figure S2: The activity of luciferase reporter system under control of mutated MREs cloned from metabolic genes. Figure S3: Western blot analysis of proteins encoded by metabolic genes in RCC cells transfected with predicted miRNA mimics or scrambled non-targeting control oligonucleotide. Figure S4: The expression of miR-103a-3p in tissue samples. File S1: Criteria used for selection of miRNAs targeting genes involved in metabolic regulation. (DOCX). File S2: The expression of NFAT5 correlates with expression of the predicted regulatory miRNAs in cancers.
