# Supplementary material for: MicroRNA-Mediated Metabolic Reprograming in Renal Cancer
Source: Cancers (Basel). 2019 Nov 20;11(12):1825. doi: 10.3390/cancers11121825 (PMC6966432; doi:10.3390/cancers11121825)
Supplement: Supplementary file 1 [file cancers-11-01825-s001.zip › cancers-565117.pdf]

cancers

MDPI

Article

Arl13b Regulates Breast Cancer Cell Migration and Invasion by Controlling Integrin-Mediated Signaling

Cristina Casalou [1,†,*], Alexandra Faustino [1,2,†], Fernanda Silva [1,3], Inês C. Ferreira [1], Daniela Vaqueirinho [1], Andreia Ferreira [1], Pedro Castanheira [1,2], Teresa Barona [1], José S. Ramalho [1], Jacinta Serpa [1,3,†], Ana Félix [1,3,†] and Duarte C. Barral [1,*]

[1] CEDOC, NOVA Medical School | Faculdade de Ciências Médicas, Universidade NOVA de Lisboa, Lisboa postcode, Portugal; alexandra.faustino@nms.unl.pt (A.F.), fernanda.silva@nms.unl.pt (F.S.), ines.c.ferreira@nms.unl.pt (I.C.F.), daniela.vaqueirinho@irb.usi.ch (D.V.), andreia.ferreira@nms.unl.pt (A.F.), pedro.castanheira@nms.unl.pt (P.C.), teresa.barona@nms.unl.pt (T.B.), jose.ramalho@nms.unl.pt (J.S.R.), jacinta.serpa@nms.unl.pt (J.S.), ana.felix@nms.unl.pt (A.F.)

[2] ProRegeM—PhD Program in Mechanisms of Disease and Regenerative Medicine, Lisboa postcode, Portugal

[3] Instituto Português de Oncologia de Lisboa Francisco Gentil (IPOLFG), Lisboa postcode, Portugal

† These authors contributed equally to this work.

* Correspondence: cristina.casalou@nms.unl.pt (C.C.), duarte.barral@nms.unl.pt (D.C.B.)

Received: 18 July 2019; Accepted: 26 September 2019; Published: date

Abstract: Breast cancer is the first cause of cancer-related mortality among women worldwide, according to the most recent estimates. This mortality is mainly caused by the tumor’s’ ability to form metastases. Cancer cell migration and invasion are essential for metastasis and rely on the interplay between actin cytoskeleton remodeling and cell adhesion. Therefore, understanding the mechanisms by which cancer cell invasion is controlled may provide new strategies to impair cancer progression. We investigated the role of the GTP-binding protein Arl13b in breast cancer cell migration and invasion in vitro, using breast cancer cell lines and in vivo, using mouse orthotopic models. We show that Arl13b silencing inhibits breast cancer cell migration and invasion in vitro, as well as cancer progression in vivo. We also observed that Arl13b is upregulated in breast cancer cell lines and patient tissue samples. Moreover, we found that Arl13b localizes to focal adhesions (FAs) and interacts with β3-integrin. Upon Arl13b silencing, β3-integrin cell surface levels and FA size are increased and integrin-mediated signaling is inhibited. Therefore, we uncover a role for Arl13b in breast cancer cell migration and invasion and provide a new mechanism for how *ARL13B* can function as an oncogene, through the modulation of FA turnover.

Keywords: integrins; actin cytoskeleton; cancer progression; Arl proteins; cell-extracellular matrix adhesion

Commented [m1]: Please carefully check the accuracy of names and affiliations.

Commented [m2]: please add

Commented [m3]: please check all authors’ emails

Commented [m4]: modified, please confirm

Commented [m5]: please confirm the order of Correspondence emails

Commented [m6]: Please define all abbreviations the first time they appear in the abstract, the main text, and the first figure or table caption.

1. Introduction

Cell migration plays a crucial role in physiological processes such as embryogenesis, immune responses and tissue repair. Moreover, cell migration dysregulation is implicated in various pathologies, like vascular disease, chronic inflammation and cancer [1]. Indeed, cancer cells subvert the machinery that regulates cell migration and invasion to escape the primary tumor site and form local and distant metastases, which are the leading cause of cancer–related deaths [2]. Therefore, targeting regulators of cell migration and invasion and understanding the mechanisms by which they control these processes may provide new strategies to delay cancer progression and impair metastasis.

Cell migration is a multistep process that requires spatiotemporal coordination of actin cytoskeleton remodeling and cell adhesion [1,3]. Moreover, cell migration relies on the driving force

generated by actin polymerization, which allows the extension of protrusions such as lamellipodia and filopodia at the cell's leading edge. These protrusions are then stabilized by the adhesion to the extracellular matrix (ECM) mediated by FAs, which link the actin cytoskeleton to the ECM via integrins [1,3]. Finally, actomyosin-generated forces allow the forward movement of the cell, while FAs at the cell's trailing edge are disassembled [1,3]. The spatial organization and turnover of FAs is controlled by integrin trafficking [4]. Moreover, increasing evidence indicates that membrane traffic is frequently subverted by cancer cells to enhance tumorigenesis. In fact, many cancers exhibit changes in the expression of membrane traffic regulators [5,6]. This raises the need to understand how the membrane traffic machinery is subverted by cancer cells in order to design new therapeutic approaches to impair tumorigenesis.

Members of the ADP-ribosylation family (Arf) family of GTP-binding (G) proteins regulate membrane traffic and actin cytoskeleton remodeling, being essential for several cellular functions, including cell spreading and migration [6,7]. This family is composed by 6 Arf proteins, 21 Arf-like (Arl) proteins, 2 Secretion-associated and Ras-related (Sar) proteins and Tripartite motif (Trim)23 [8]. Interestingly, Arfs and Arf GTPase-activating proteins (GAPs) have been shown to regulate cell adhesion by modulating the localization of FA components, the trafficking of integrins and actin cytoskeleton remodeling [6,9]. Furthermore, upregulation of Arf1, Arf4 and Arf6, as well as the Arf GAPs AGAP2, ASAP1, ASAP3 and GIT1 correlates with enhanced migration, invasion and metastasis of breast cancer cells [6,10].

Commented [m7]: please define

Arl13b was originally identified as a regulator of ciliogenesis and Sonic hedgehog (Shh) signaling [11,12]. Moreover, recent studies showed that Arl13b promotes gastric carcinogenesis, cell migration and invasion [13], as well as medulloblastoma formation [14]. However, the molecular mechanism by which Arl13b regulates cancer cell migration and invasion is not understood. We have established a previously unidentified role for Arl13b in the regulation of endocytic recycling traffic [15] and cell migration in non-pathological conditions [16]. Moreover, we reported that Arl13b regulates cell migration through actin cytoskeleton remodeling via the interaction with the non-muscle Myosin IIA (NMIIA) [16]. Therefore, we hypothesized that Arl13b regulates migration and invasion of cancer cells through actin cytoskeleton-related mechanisms.

We decided to study this question in breast cancer, which is the most frequent type of cancer in women, with 2.09 million cases estimated worldwide in 2018 and the prime cause of cancer-related deaths among women [17]. Here, we provide evidence that Arl13b positively regulates breast cancer progression. We observed that Arl13b silencing impairs breast cancer cell migration and invasion in vitro and tumor growth and metastasis in vivo. Moreover, we found that Arl13b interacts with β3-integrin, localizes to FAs and regulates stress fiber (SF) formation and FA size. Furthermore, we observed that Arl13b positively regulates the integrin-mediated signaling involved in the control of FA dynamics. Thus, our observations suggest that Arl13b is involved in cell-ECM adhesion by promoting FA turnover. Furthermore, we show that Arl13b is upregulated in breast cancer cell lines and tissues. Thus, our study reinforces previous evidence for the involvement of Arl13b in cancer and suggests that Arl13b is subverted by breast cancer cells to become more motile and invasive, with potential to be targeted for therapeutic purposes.

2. Results

2.1. Arl13b Positively Regulates Breast Cancer Cell Migration and Invasion

We previously showed that Arl13b regulates migration of mouse fibroblasts in vitro and zebrafish neural crest cells in vivo [16]. To investigate whether Arl13b could confer enhanced migratory and invasion capacities to breast cancer cells, we started by assessing the effect of Arl13b silencing in migration and invasion of a highly invasive breast cancer cell line (MDA-MB-231), by using two distinct short hairpin (sh) RNAs targeting Arl13b (E4 and E6).

As controls, cells were transduced with lentiviruses encoding a non-targeting shRNA (shRNA Mission) or the lentiviral vector (shRNA Empty). The efficiency of the shRNA-mediated silencing was confirmed by RT-qPCR and immunoblotting (Figures S1A and B). Since we and others observed

that Arl13b silencing impairs cell growth (Figure S1C; [13,14,18]), we used mitomycin C to inhibit cell proliferation and exclude proliferation interference in migration and invasion assays lasting for more than 8 hours. Mitomycin C efficiency was confirmed by monitoring the number of viable cells (Figure S1D).

Cell migration was assessed by scratch assaysand transwell assays. In both assays, we found that Arl13b silencing reduces by more than 50% the capacity of MDA-MB-231 cells to migrate (Figure 1A and B). We then used transwell invasion assays and observed a significant impairment of the ability of MDA-MB-231 cells to invade through matrigel-coated filters, when Arl13b is silenced (Figure 1C).

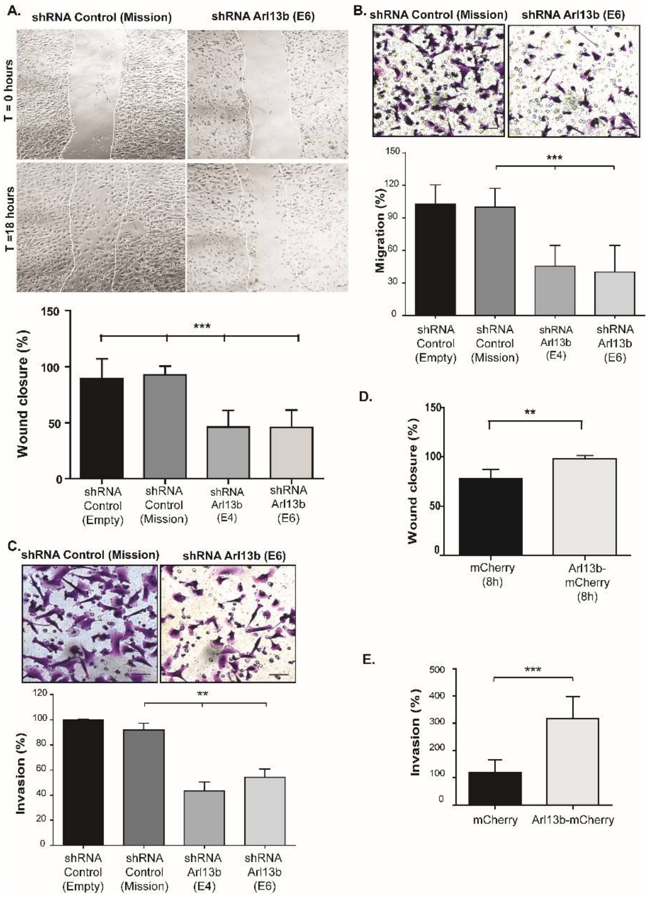

Figure 1. Arl13b affects breast cancer cell migration and invasion. (**A**) Arl13b-silenced (shRNA E4 and E6) and control (shRNA Mission and Empty vector) MDA-MB-231 cells were grown to confluency, grown on plates pre-coated with 10 μg/mL fibronectin in PBS, treated with mitomycin C and scratched with a pipette tip. Cell migration was induced by 10% FBS addition to serum-free medium. Representative images at 0- and 18-hours post-scratching are shown for control and Arl13b-silenced cells (Mission and E6, respectively). Quantitative analysis of gap ("wound") closure was performed after 18 hours as described in Materials and Methods. Error bars represent mean ± SD (n = 3). Scale bars, 20 μm. *** $p < 0.001$ (one-way ANOVA). (**B** and **C**) Arl13b-silenced or control MDA-MB-231 cells in serum-free medium were placed into the upper chamber of 8 μm transwells without (**B**) or with (**C**) matrigel and allowed to migrate and invade. After 6 hours (**B**) or 21 hours (**C**), cells that

Commented [m8]: Please add scale bar or magnification for this subfigure A, B and C.

migrated/invaded through the transwell membrane were fixed and stained with crystal violet. Representative images are shown. Scale bars, 50 μm. Cells from at least 10 randomly-chosen fields were counted. For each condition, the percentage of migration (**B**) and invasion (**C**) was normalized to shRNA control. Error bars represent mean ± SD ($n \geq 3$). ** $p < 0.01$ (unpaired two-tailed Student's t-test, Mann-Whitney). (**D**) Scratch assay was performed as in (**A**) with MDA-MB-231 cells expressing Arl13b-mCherry or mCherry (control). The percentage of gap ("wound") closure was measured after 8 hours. Error bars represent mean ± SD ($n = 3$). ** $p < 0.01$ (**E**) Cells expressing Arl13b-mCherry or mCherry were induced to invade and migrate as in (**C**). Invasion (%) was determined in at least three independent experiments as in (**C**) and error bars represent mean ± SD. *** $p < 0.001$ (unpaired two-tailed Student's *t*-test, Mann-Whitney).

Next, we tested the effect of Arl13b overexpression in breast cancer cell migration and invasion and observed that it enhances both migration (Figure 1D and Figure S2A) and invasion (Figure 1E and Figure S2B) of MDA-MB-231 cells. A similar effect in cell migration was observed when we overexpressed Arl13b in the weakly invasive MCF-7 breast cancer cell line (Figure S2C and D). Together, our results indicate that Arl13b positively regulates breast cancer cell migration and invasion in vitro.

2.2. Arl13b Regulates Focal Adhesion Stability in Breast Cancer Cells

To investigate the mechanism by which Arl13b promotes cell migration and invasion and knowing that Arl13b interacts with actin [15], we evaluated its involvement in actin cytoskeleton remodeling in breast cancer cells. We observed that endogenous Arl13b colocalizes with filamentous (F)-actin in MDA-MB-231 cells, particularly in SFs, lamellipodia and filopodia at the ventral plane of the cell and in filopodia and CDRs at the dorsal surface of the cell (Figure S3), confirming previous results obtained in fibroblasts where Arl13b-GFP was overexpressed [16]. SFs are the major contractile structures in non-muscle cells, being NMII one of their major components [19]. We previously described that NMIIA is an Arl13b effector in mouse fibroblasts [16]. Now, we confirmed that NMIIA interacts with Arl13b in MCF-7 and MDA-MB-231 breast cancer cells preferentially when the cell lysates are loaded with GTPγS, a non-hydrolysable form of GTP, as compared with excess GDP (Figure S4 A and B). NMII-induced actin cytoskeletal tension is required for FA growth and has a key role in cell adhesion and contraction [3,19]. Several Arfs and Arf GAPs have been shown to play a role in the establishment of FAs [6,9]. Therefore, we assessed whether Arl13b also localizes to FAs and has a role in their formation and/or stability. For this, we evaluated Arl13b colocalization with endogenous Vinculin, a component of FAs. Indeed, we observed that Vinculin partially co-localizes with Arl13b-mCherry (Figure 2A) and endogenous Arl13b (Figure 2B), in MDA-MB-231 cells. Moreover, by live cell imaging, we observed that Arl13b-EGFP is recruited to FAs and colocalizes with DsRed-tagged Paxillin, another component of FAs (Video S1).

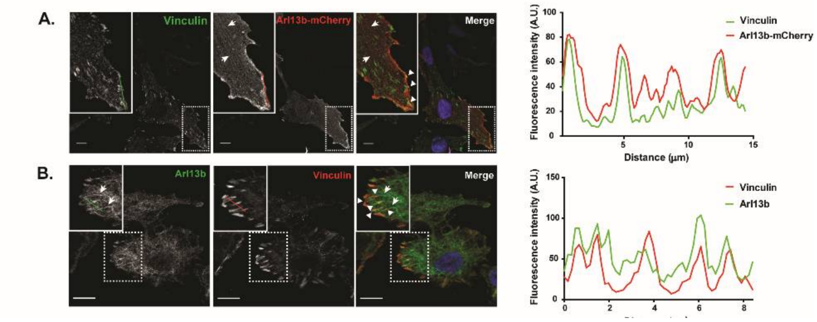

Figure 2. *Cont.*

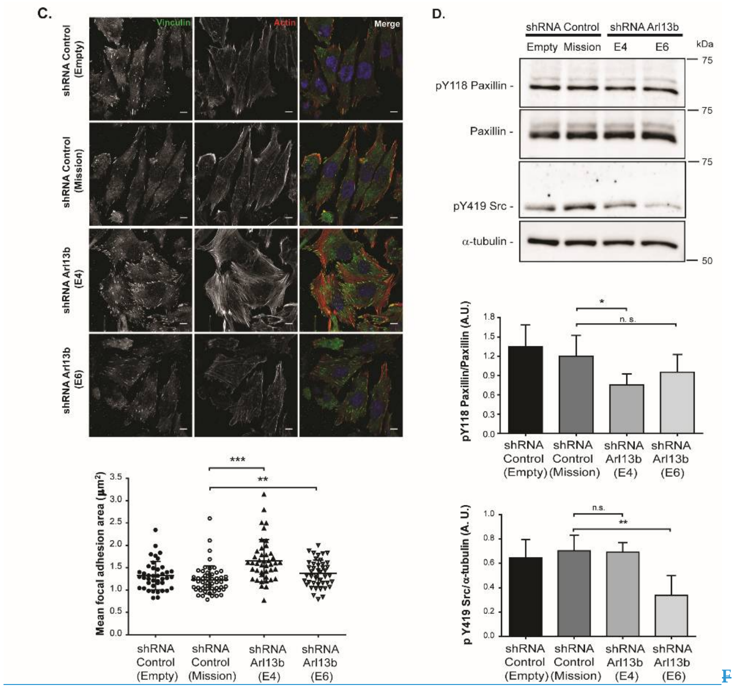

Figure 2. Arl13b localizes to and regulates focal adhesions size and integrin-mediated signaling in breast cancer cells. (**A**) MDA-MB-231 breast cancer cells stably expressing Arl13b-mCherry, grown on coverslips pre-coated with 10 µg/mL fibronectin in DMEM with 10% FBS, were immunostained for Vinculin (green). Zoom-ins of the indicated sections are shown in the insets. Arrowheads indicate Arl13b localization in focal adhesions and arrows indicate Arl13b with filamentous distribution, consistent with SFs. Scale bars (**B**) MDA-MB-231 cells were immunostained with anti-Arl13b (green) and anti-Vinculin (red) antibodies. Arrowheads indicate co-localization of endogenous Arl13b with Vinculin at focal adhesions and arrows indicate Arl13b with filamentous distribution. Scale bars, 10 µm. Panels on the right represent the intensity profiles of Vinculin and Arl13b-mCherry (**A**) and Vinculin and endogenous Arl13b (**B**), obtained using the plot profile function of ImageJ, along the green and red lines represented in the images of the separate channels. (**C**) Arl13b-silenced (shRNA E4 and shRNA E6) or control (shRNA Mission and Empty) MDA-MB-231 breast cancer cells, grown on coverslips with 10 µg/mL fibronectin in DMEM with 10% FBS, were stained for F-actin with phalloidin (red), Vinculin (green) and DAPI (blue) to detect nuclei. The Vinculin staining was used to measure FA size in at least 10 cells in each condition per experiment, as described in Material and Methods. Error bars represent mean ± SD ($n = 3$). ** $p < 0.01$; *** $p < 0.001$ (unpaired two-tailed Student's t-test, Mann-Whitney). Scale bars, 10 µm. (**D**) Expression levels of pY118 Paxillin, total Paxillin and pY419 Src were determined in Arl13b-silenced (shRNA E4 and E6) and control (shRNA Empty and Mission) MDA-MB-231 cells, grown on wells coated with 10 µg/mL fibronectin in DMEM with 10% FBS, by immunoblotting. The levels of pY118 Paxillin were determined relative to total Paxillin levels, both normalized to the levels of the loading control -tubulin. The levels of pY419 Src were determined relative to the loading control α-tubulin. Error bars represent mean ± SD ($n \geq 3$). ** $p < 0.01$; *$p < 0.05$; n.s., non-significant (unpaired two-tailed Student's t-test, Mann-Whitney). A.U., arbitrary units.

Commented [m9]: please add

Commented [m10]: Missing anything?

Next, we assessed if Arl13b silencing influences FA size. For this, Arl13b-silenced and control MDA-MB-231 cells were immunostained for Vinculin to detect FAs. We observed that Arl13b-silenced cells show an increase in FA mean size when compared with control cells (Figure 2C). Also,

by examining phalloidin staining, we detected an altered pattern of SFs in Arl13b-silenced cells (Figure 2C).

Supporting the altered SF formation, we found that NMIIA mRNA and protein expression levels, are increased in Arl13b-silenced cells relative to control cells (Figure S4 C and D). Thus, our results suggest that Arl13b negatively regulates NMIIA expression and SF formation, therefore affecting FA growth in breast cancer cells.

FAs disassembly is regulated by activation of protein tyrosine kinases such as focal adhesion kinase and Src and phosphorylation of FA proteins such as Paxillin [20]. Moreover, Zaidel-Bar et al. demonstrated that non-phosphorylatable Paxillin stabilizes adhesion sites [21]. Therefore, we measured the levels of phosphorylated Paxillin (Y118) and the activation levels of Src (pY419) in MDA-MB-231 cells. We found a decrease in pY118 Paxillin levels upon Arl13b silencing, using both Arl13b shRNAs and in pY419 Src, upon stronger Arl13b silencing obtained with E6 hairpin (Figure 2D). These results suggest that the formation of larger FAs in Arl13b-depleted cells may result from an inhibition of integrin-mediated signaling, which regulates FA turnover.

2.3. Arl13b Interacts with and Negatively Regulates β3-Integrin Levels at the Cell Surface of Breast Cancer Cells

Integrin binding to the ECM is the first step in cell adhesion and precedes FA assembly [22,23]. Given the increase in size and stability of FAs observed in Arl13b-silenced cells, we investigated the effect of Arl13b silencing in β3-integrin surface levels in MDA-MB-231 cells. We observed a significant increase in β3-integrin surface levels upon Arl13b silencing, relative to cells transduced with controls vectors (Figure 3A).

To assess the specificity of this effect, we expressed Arl13b-mCherry in MDA-MB-231 cells silenced for Arl13b. We observed that β3-integrin cell surface levels are rescued to control levels upon Arl13b overexpression, confirming that the increase in β3-integrin surface levels observed in Arl13b-silenced cells is not an off-target effect of the shRNAs used (Figure 3B). Interestingly, we also found that β3-integrin co-immunoprecipitates with Arl13b (Figures 3C and D). Therefore, these results suggest that Arl13b interacts with β3-integrin and negatively regulates its surface levels, supporting the observation of larger and more stable FAs in Arl13b-depleted cells.

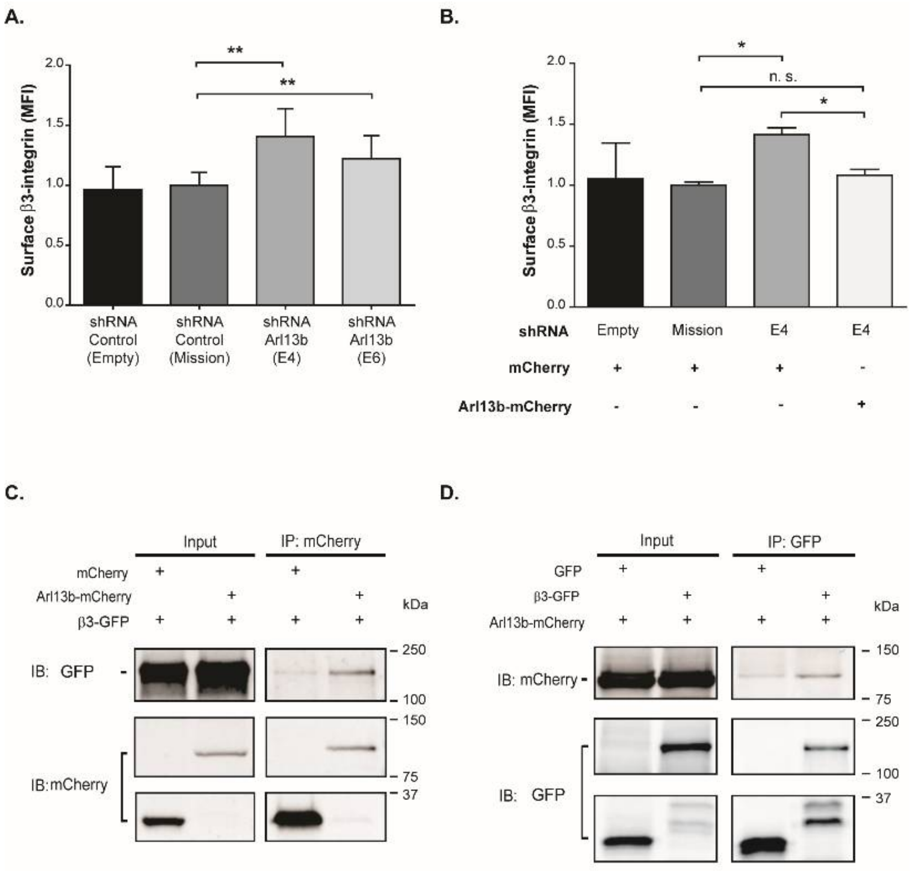

Figure 3. Arl13b interacts with and regulates β3-integrin cell surface levels in breast cancer. (**A**) β3-integrin surface levels in Arl13b-silenced (shRNA E4 and E6) and control (shRNA Empty and Mission) MDA-MB-231 cells were analyzed by flow cytometry. For each condition, the median fluorescence intensity (MFI) of β3-integrin expression in propidium iodide-negative cells was normalized to shRNA control. Error bars represent mean ± SD ($n \geq 4$). ** $p < 0.01$ (unpaired one-tailed Student's t-test, Mann-Whitney). (**B**) MFI of β3-integrin cell surface expression in Arl13b-mCherry- (rescue) or mCherry-positive cells was determined in cells silenced for Arl13b (shRNA E4) or control cells (shRNA Empty and Mission). Error bars represent mean ± SD ($n = 2$). * $p < 0.05$; n.s., non-significant (unpaired one-tailed Student's t-test, Mann-Whitney). (**C** and **D**) Total cell extracts of MDA-MB-231 breast cancer cells stably expressing Arl13b-mCherry or mCherry and transfected with β3-integrin-GFP or GFP were immunoprecipitated (IP) with anti-mCherry (**C**) or anti-GFP (**D**) antibodies. Immunoprecipitates were analyzed by immunoblotting (IB) using anti-GFP (**C**) or anti-mCherry (**D**) antibodies. The efficiency of immunoprecipitation was assessed by IB with anti-mCherry (**C**) or anti-GFP (**D**) antibodies. Experiments were independently performed twice.

2.4. *Arl13b Positively Regulates Breast Tumorigenesis In Vivo*

Since we established Arl13b as a positive regulator of breast cancer cell migration and invasion in vitro, we investigated if Arl13b plays role in breast tumorigenesis in vivo, using a murine orthotopic model of breast cancer. For that, MDA-MB-231 cells stably silenced for Arl13b or control cells were xenografted into the mammary fat pads of immunocompromised BALB/c-SCID mice. Strikingly, we observed that tumors formed in mice injected with Arl13b-depleted cells are

significantly smaller when compared with primary tumors formed in mice injected with control cells (Figure 4A). Furthermore, analysis of lung tissue sections revealed a significantly reduction in the number of lung metastases in mice injected with Arl13b-silenced cells, compared with mice injected with control cells (Figure 4B). We then investigated if the overexpression of Arl13b enhances tumorigenesis in vivo. For this, we injected in the fat pad of BALB/c-SCID mice MDA-MB-231 cells stably overexpressing Arl13b-mCherry or mCherry. We found that Arl13b overexpression leads to a significant increase in the size of breast tumors (Figure 4C) and abundant metastases in the lungs (Figure 4D). Collectively, these results indicate that Arl13b promotes both primary breast tumor formation and metastatic dissemination in vivo.

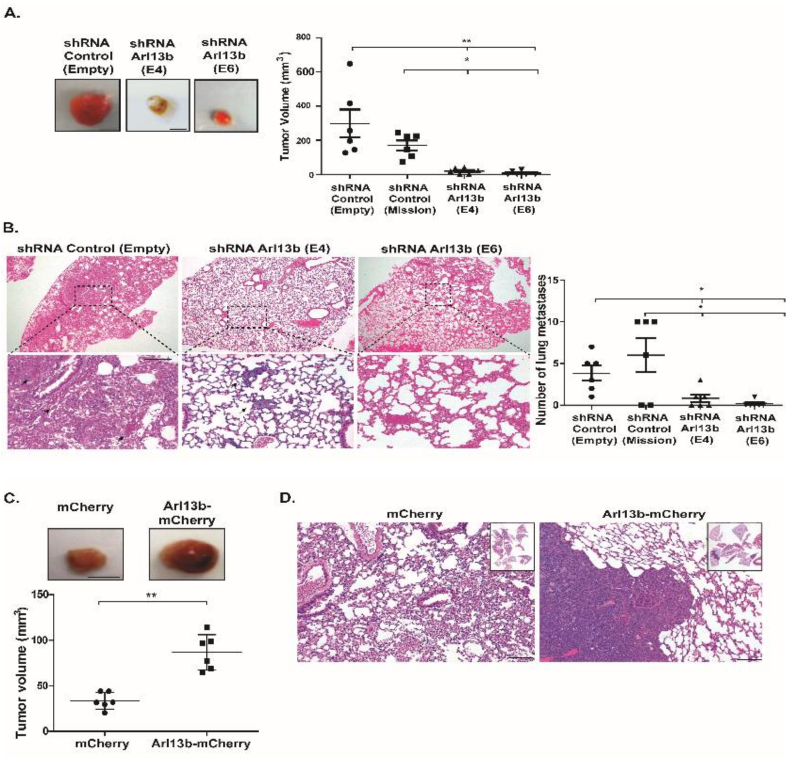

Figure 4. Arl13b positively regulates breast cancer development in vivo. Arl13b-silenced (shRNA E4 and E6) or control (shRNA Empty and Mission) MDA-MB-231 cells were injected in the fat pad of BALB/c-SCID mice. (**A**) After 6–8 weeks, primary tumors were excised and measured, as described in Materials and Methods. Representative images of the primary tumors are shown. Error bars represent mean ± SD (n = 6 for each condition). ** $p < 0.01$, * $p < 0.05$ (one-way ANOVA). Scale bars, 0.5 cm. (**B**) Representative images of lung sections (H&E staining) of mice injected with Arl13b-silenced or control MDA-MB-231 cells. Black arrows indicate metastases in lung sections. Scale bars, 100 μm. Metastases were quantified in lung sections of mice injected with Arl13b-silenced (shRNA E4 and E6) and control (shRNA Empty and Mission) cells. Error bars represent mean ± SD. * $p < 0.05$ (one-way ANOVA). (**C**) MDA-MB-231 cells overexpressing Arl13b-mCherry or mCherry (control) were injected in the fat pad of BALB/c-SCID mice. After 4–6 weeks, primary tumors were surgically removed and measured to determine tumor volume. Error bars represent mean ± SD. Experiments were independently performed twice (n = 6). ** $p < 0.01$ (unpaired one-tailed Student's t-test, Mann-Whitney). Scale bars, 0.5 cm. (**D**) Representative images of lung sections (H&E staining) of mice

injected with MDA-MB-231 cells overexpressing Arl13b-mCherry or mCherry. (insets show lungs in low magnification). Scale bars, 100 μm.

2.5. Arl13b Expression is Upregulated in Breast Cancer Cell Lines and Tissue Samples

To further ascertain the relevance of Arl13b in breast cancer progression, we assessed Arl13b mRNA and protein expression levels in different breast cancer cell lines with distinct invasive capacities [24]. We found that the weakly invasive breast cancer cell lines BT-474 and MCF-7, and the highly invasive line MDA-MB-231 express significantly higher levels of Arl13b mRNA when compared with the in situ breast cancer cell line MCF10DCIS.com (Figure 5A). Moreover, at the protein level we observed that MCF-7 and MDA-MB-231 cell lines express markedly higher levels of Arl13b than MCF10DCIS.com (Figure 5B). Therefore, Arl13b expression levels positively correlates with the invasive capacity of breast cancer cells.

Next, we examined whether the expression of Arl13b is modulated in tissue samples derived from breast cancer patients by comparing mRNA and protein levels in primary breast carcinomas and normal adjacent tissue samples (Figure 5C–E). For this, freshly-collected breast carcinoma samples and their paired adjacent normal tissues (Table S3) were analyzed by qRT-PCR for Arl13b expression levels. We observed that Arl13b mRNA expression levels are increased in more invasive tumors, being statistically significantly elevated in grade II, when compared with grade I breast carcinomas or the paired adjacent tissues (Figure 5C). Additionally, the analysis of another dataset of, frozen breast carcinoma samples and adjacent normal breast tissues (Table S4) by immunoblotting shows that Arl13b protein levels are statistically significantly upregulated in breast carcinomas, when compared to adjacent normal breast tissues (Figure 5D and E). Furthermore, immunohistochemical analysis showed that Arl13b expression is detected mainly in ductal epithelial cells of normal adjacent breast tissues (Figure 5F; black arrows), whereas carcinoma cells are strongly stained for Arl13b (Figure 5F; arrowheads). This was confirmed with different breast carcinoma samples and normal breast tissue derived from mammary reductions, as well as a different anti-Arl13b antibody (Figure S5).

Together, our results suggest that Arl13b is subverted by breast carcinomas, which upregulate its expression to become more migratory and invasive and that this might be achieved by controlling FA turnover through modulation of integrin-mediated signaling and cell-ECM adhesion (Figure 6).

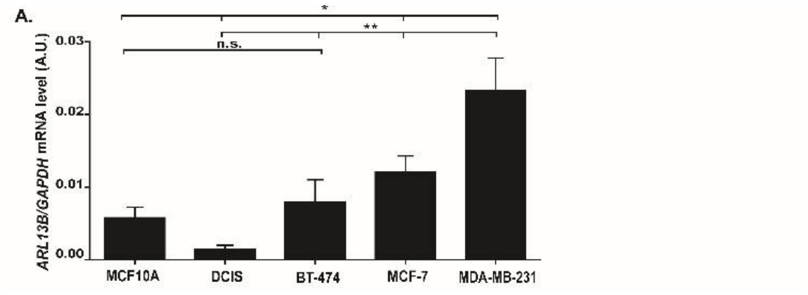

Figure 5. *Cont.*

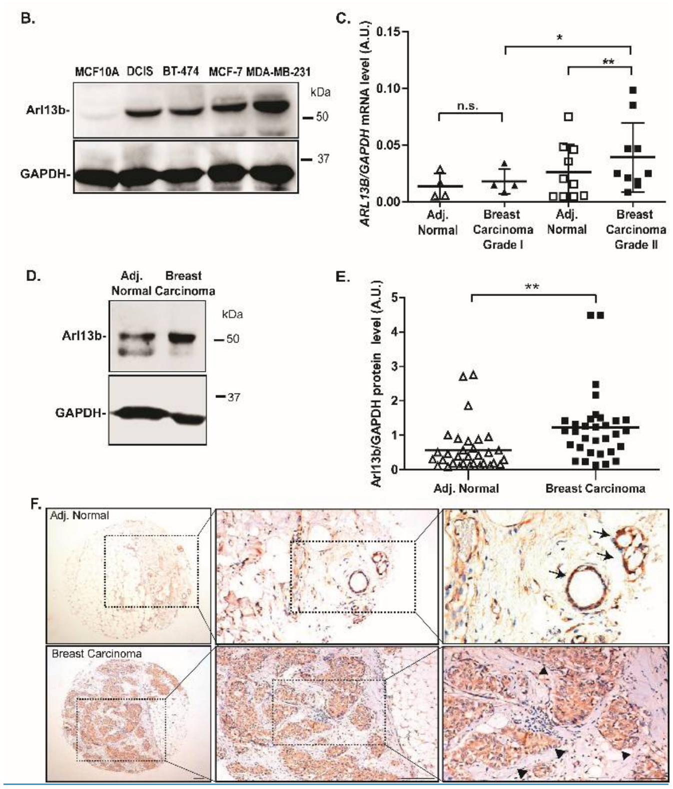

Figure 5. Arl13b expression is upregulated in breast cancer cells and tissues. Arl13b mRNA (**A**) and protein (**B**) expression levels in non-tumorigenic (MCF10A), in situ breast cancer cell line MCF10DCIS.com (DCIS), and invasive breast cancer cell lines BT-474, MCF-7 and MDA-MD-231. Arl13b expression is represented relative to the levels GAPDH. Error bars represent mean ± SD (n = 3). * $p < 0.05$; ** $p < 0.01$; n.s., non-significant (unpaired one-tailed Student's t-test, Mann-Whitney). A.U., arbitrary units. (**C**) Arl13b mRNA levels were determined in invasive ductal breast carcinomas of grades I and II and compared with the levels in adjacent parenchyma normal breast tissue samples by qRT-PCR. (**D**) Arl13b protein expression levels were determined by immunoblotting in breast carcinoma samples and compared with the levels in adjacent normal breast tissue samples. Representative pair is shown, where GAPDH was used as a loading control. Error bars represent mean ± SD (n = 14). ** $p < 0.01$, *$p < 0.05$, n.s.-non-significant (paired, Student's t-test). A.U., arbitrary units (**E**) Arl13b/GAPDH protein expression ratio was determined in adjacent normal breast tissue and invasive breast carcinomas. Error bars represent mean ± SD (n = 32). ** $p < 0.01$ (paired Student's t-test). A.U., arbitrary units. (**F**) Representative Arl13b immunohistochemistry staining of adjacent normal and invasive carcinoma NOS paired (pT1pN1, G2) are shown. Positivity is seen in normal epithelial cells of the acini (arrows), whereas negativity is observed in the stroma. Intense positive tumor cells (arrowheads) are observed in carcinoma tissues. Scale bars, 100 μm.

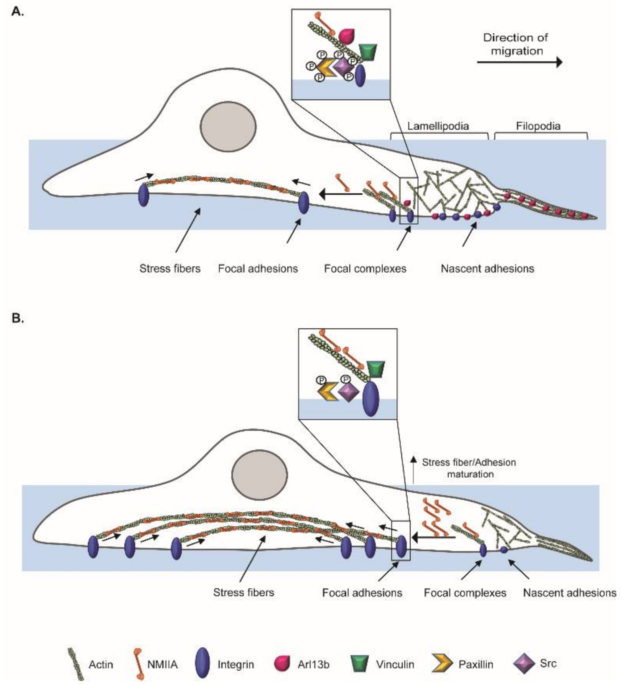

Figure 6. Schematic of proposed mechanism for Arl13b regulation of breast cancer cell migration and invasion. (**A**) In breast cancer cells, Arl13b localizes to actin-rich structures such as filopodia, lamellipodia and SFs. Arl13b is also recruited to FAs, since it colocalizes with Paxillin and Vinculin and interacts with β3-integrin. (**B**) When Arl13b is silenced, the increased NMIIA levels can contribute to the formation of more SFs and FA growth through tensile forces generated by Myosin II-driven contraction of SFs. This increases β3-integrin levels at the cell surface and FA size, as we observed. Furthermore, Paxillin and Src phosphorylation are decreased in Arl13b-silenced cells. These phenotypes suggest that breast cancer cells depleted for Arl13b have defects in FA disassembly that contribute to the impairment in migration and invasion.

3. Discussion

Breast cancer is the most frequent type of cancer in women worldwide and the related deaths result largely from metastatic spread of the primary tumor. In this study, we describe a new role for *ARL13B* as an oncogene in breast cancer progression and provide a possible mechanism by which this G protein regulates cancer cell migration and invasion. We demonstrate that Arl13b is involved in actin SF formation and the control of FA disassembly through integrin signaling, resulting in a negative regulation of FA size and stability, as well as β3-integrin cell surface levels. This strongly suggests a role for Arl13b in cell-ECM adhesion (Figure 6). Strikingly, by analyzing breast cancer tissue samples, we found that Arl13b is upregulated in breast carcinomas when compared with the expression in adjacent normal breast. This extends recent reports demonstrating a role for Arl13b in gastric tumorigenesis and medulloblastoma formation [13,14]. Thus, *ARL13B* can be considered an oncogene with potential to be targeted in anticancer therapies.

Cell migration relies on spatiotemporal coordination of actin cytoskeleton remodeling and cell-ECM adhesion. Arl13b could link these two processes since it binds to actin via NMIIA and plays a role in actin cytoskeleton remodeling [16]. Indeed, we observed colocalization of endogenous Arl13b with actin in structures such as lamellipodia, filopodia, SFs and CDRs of breast cancer cells. Besides actin, NMIIA is a major component of SFs, which we previously identified as an Arl13b effector [16].

Interestingly, we observed that Arl13b interacts with NMIIA in a GTP-dependent manner in breast cancer cells. Moreover, we found an inverse correlation between Arl13b and NMIIA expression. As to whether this phenotype results from an Arl13b-dependent regulation of NMIIA expression levels, protein degradation or another mechanism, needs to be further explored. Furthermore, the increased expression of NMIIA may lead to the enhanced formation of SFs. Although the function of SFs in cell migration is not fully established, our results reinforce studies showing that SFs are more prominent in non-migrating cells, while highly motile cells show fewer, thinner and more dynamic actin bundles [25,26].

SFs can regulate cell-ECM adhesion by controlling FA size. Supporting the observation of an altered pattern of SFs in Arl13b-silenced cells, we observed that Arl13b depletion leads to larger FAs. Upon the engagement of integrins with the ECM, integrin signaling at the FA complex is activated. The activation of the FAK/Src complex and phosphorylation of Paxillin have an established role in FA turnover [20,21]. When Arl13b is silenced, activation of Src is attenuated and a decrease in Paxillin is observed. This suggests that the increased in FA size observed in Arl13b-silenced cells may result from defects in the signaling involved in FA disassembly Consistent with a role for Arl13b in FA dynamics, we also observed that Arl13b localizes to these structures in fixed and live cells. Since cell migration requires efficient assembly and disassembly of FAs [27], we propose that a decrease in the disassembly rate of FAs in Arl13b-silenced cells contributes to an abnormal cell-ECM adhesion and decreased cell migration.

FA maturation and cell-ECM adhesion are not only controlled by actomyosin dynamics but also by the integrin trafficking [4]. Indeed, in growth factor-stimulated cell migration, β3-integrin can be endocytosed through CDR-mediated macropinocytosis and then trafficked through the endocytic recycling compartment to newly-established FAs on the ventral surface, at the leading edge of migrating cells [28]. We observed that Arl13b interacts with β3-integrin and downregulates its cell surface levels. Our group has also shown that Arl13b is required for CDR formation [16]. Therefore, it is tempting to speculate that Arl13b controls β3-integrin surface levels by regulating the internalization and/or recycling of integrins through CDRs.

Arl13b has a well-established role in the trafficking of ciliary proteins and ciliogenesis [11,12]. Notably, we reported its interaction with actin through NMIIA, its localization to actin-rich structures [16] and now the interaction of Arl13b with β3-integrin and co-localization with FA components, namely Vinculin and Paxillin. Interestingly, it is now appreciated that many ciliary proteins have other localizations besides primary cilia and that they can play a role in the formation of polarized cellular structures [29]. Furthermore, it was described that FA proteins associate with basal bodies of ciliated cells and form ciliary adhesion complexes that interact with the actin cytoskeleton [30]. Therefore, Arl13b functions in primary cilia and FA dynamics further suggest that primary cilia and FAs might share protein components that are recruited to the appropriate location depending on the cellular context or cell transformation status.

Our results also confirmed the role of Arl13b in cell growth suggested by other studies [13,14,18]. Indeed, mice injected with Arl13b-depleted breast cancer cells hardly develop primary tumors and when they do, these are very small. In contrast, mice injected with Arl13b-overexpressing breast cancer cells develop large tumors. Although the impairment in the formation of cancer metastases in mice injected with Arl13b-depleted breast cancer cells is consistent with the in vitro studies pointing to Arl13b being essential for cancer cell migration and invasion, further studies have to be performed where primary tumors are allowed to develop and only then Arl13b is depleted, by using an inducible system. This would allow the uncoupling of the effects of Arl13b depletion in cell proliferation and migration/invasion.

Importantly, breast cancer is the third type of cancer whose progression or formation has been shown to be promoted by Arl13b and we also have evidence that the same occurs in colon cancer (our unpublished results). Indeed, recently published studies on gastric cancer and medulloblastoma proposed a Shh signaling-dependent mechanism [13,14]. However, breast cancer is a type of cancer where tumor cells show a decrease or loss of primary cilia [31–33], contrary to medulloblastoma and gastric cancer cells, where cilia are present [34,35] and cilia-dependent Shh signaling can then play

an important role. Therefore, the mechanisms governing Arl13b-induced carcinogenesis might differ between different cancer types. Nevertheless, we cannot exclude the influence of non-canonical Shh signaling, which is cilia-independent, in breast carcinogenesis. Thus, future studies should investigate the contribution of Arl13b-regulated actin remodeling and FA dynamics, as well as canonical and non-canonical Shh signaling for carcinogenesis.

4. Materials and Methods

4.1. Cell Culture

Cell culture media, supplements and antibiotics were purchased from GIBCO (city, state abbrev if USA, country). MDA-MB-231 and MCF-7 human breast adenocarcinoma cell lines (obtained from ATCC, city, state abbrev if USA, country) were maintained at 37 °C and 5% CO2, in DMEM supplemented with 10% fetal bovine serum (FBS), 100 U/mL penicillin, 100 µg/mL streptomycin, 2 mM L-glutamine and 15 mM HEPES (DMEM complete medium) for up to 1 month. BT-474 human breast adenocarcinoma cell lines (obtained from ATCC) were maintained at 37 °C and 5% CO2, in RPMI supplemented with 10% fetal bovine serum (FBS), 100 U/mL penicillin, 100 µg/mL streptomycin, 2 mM L-glutamine and 15 mM HEPES. MCF10A-ER-Src cell line (MCF10A), kindly provided by F. Janody (i3S, Universidade do Porto, Porto, Portugal) [36], was grown in DMEM/F12, supplemented with 5% horse serum, 20 ng/mL epidermal growth factor (EGF), 10 µg/mL insulin, 0.5 µg/mL hydrocortisone, 100 ng/mL cholera toxin, 100 U/mL penicillin and 100 µg/mL streptomycin. MCF10DCIS.com cell line (DCIS) was purchased from the Animal Model & Therapeutic Evaluation Core (AMTEC, City, State if any, Country) Barbara Ann Karmanos Cancer Institute (City, State if any, Country), Wayne State University (City, State if any, Country). DCIS cells were grown in the same media as MCF10A cells.

Commented [m11]: please add

4.2. Cell Transduction and Lentivirus Production

MDA-MB-231 or MCF-7 were plated at 1-2 × 10^5 cells/well on 6-well plates and transduced on the next day with lentiviral particles in the presence of 6–8 µg/mL polybrene (hexadimethrine bromide, Sigma-Aldrich City, State if any, Country). After 24 hours, 1.5 µg/mL puromycin, 10 µg/mL blasticidin (MDA-MB-231) or 8 µg/mL blasticidin (MCF-7) were added to select transduced cells. Cells were selected for at least 5 days before assayed. In the rescue experiment, 1 µg/mL of puromycin and 10 µg/mL of blasticidin were used. The lentiviral vectors pLKO.1 and pLenti6/V5-DEST Gateway were used to achieve stable shRNA-mediated gene silencing or overexpression, respectively. For lentivirus production, HEK-293T grown in DMEM supplemented with 10% FBS, 2 mM GlutaMAX and 15 mM HEPES were co-transfected using FuGENE (Promega, City, State if any, Country) with 1 µg of lentiviral vector, 900 ng packaging plasmid (pCMV-dR8.91 or pxPAX2) and 100 ng envelope plasmid (pCMV-VSV-G). Media containing lentiviral particles were harvested 40 or 64 hours post-transfection and stored in aliquots at 80 °C until use. The target sequences for Arl13b hairpin E4 and the negative control Mission are described elsewhere [15]. Target sequence for hairpin E6 is: 5′-CCTATATTGGTGTTGGCAAAT-3′. shRNAs were obtained from the Broad Institute RNAi Consortium (city, state abbrev if USA, country).

4.3. RNA Extraction, cDNA Synthesis and Real-Time Quantitative PCR

Total RNA was isolated from 14 samples of freshly-collected invasive ductal breast carcinoma tissues and their adjacent parenchyma tissues using TRIzol reagent (Invitrogen, City, State if any, Country), according to the manufacturer's instructions. The extracted RNA was pretreated with DNAse I (ThermoFisher, City, State if any, Country) before cDNA synthesis. In the case of cell lines, total RNA was isolated using the RNeasy Mini Kit (QIAGEN, City, State if any, Country), according to the manufacturer's instructions. RNA was used as template for cDNA synthesis using SuperScript II Reverse Transcriptase (Invitrogen), according to the manufacturer's instructions. Real-time quantitative PCR (RT-qPCR) was performed using the FastStart Essential DNA Green Master kit (Roche, City, State if any, Country) and a LightCycler 96 System (Roche), according to the

manufacturer's instructions. All Arl13b expression levels were normalized to the level of GAPDH expression, which was used as a housekeeping gene. The primer sequences used for the human genes are listed in Table S1.

4.4. Transwell Migration/Invasion and Scratch Assays

Scratch assays were performed as described elsewhere [16]. For scratch assays, confluent cell monolayers grown on plates pre-coated with 10 µg/mL fibronectin in PBS were scratched with a pipette tip and induced to migrate by adding DMEM with 10% FBS. Images were taken from each well immediately (time 0) and after 4, 8 and 21 hours. The area of cell migration was measured in 10 randomly-chosen fields using ImageJ software. Percentage of gap closure was determined as follows: [1 − (gap area at t = 4–18 hours/gap area at t = 0 hours) × 100]. For transwell migration and invasion assays, 5×10^4 cells were seeded into the upper chamber of 8 µm-pore transwells (Corning, City, State if any, Country) in DMEM with 0.5% bovine serum albumin and allowed to migrate for 6 hours or to invade for 21 hours, towards the lower chamber containing DMEM with 10% FBS. In the case of the invasion assays, cells were seeded into the upper chamber of transwells coated with Matrigel (Corning). In the case of migration assays, cells were seeded into the upper chamber of transwells with the lower side of the membrane pre-coated overnight with 10 µg/mL fibronectin in DMEM with 10% FBS. At the end of the incubation period, cells that migrated/invaded were fixed with 4% paraformaldehyde (PFA) in PBS and stained with 0.1% (w/v) crystal violet in 20% methanol for 15 minutes, whereas the cells remaining in the upper chamber of the transwells were removed with a cotton swab. Images of the cells that migrated/invaded through the membranes were taken using an Axiovert 40 (Zeiss, City, State if any, Country) microscope equipped with a digital camera (AxioCam MRc, City, State if any, Country) and ZEN 2 software (blue edition, City, State if any, Country). The number of cells that migrated/invaded in at least 10 randomly-selected fields in duplicate for each condition were counted using ImageJ software. The number of cells that migrated/ invaded through the transwell membrane were represented as the percentage of migration/invasion relative to control cells, which was considered 100%.

4.5. Immunofluorescence

Cells were grown for 24–48 hours on glass coverslips, coated with 10 µg/mL fibronectin in DMEM with 10% FBS when indicated, washed with PBS and fixed in 4% PFA in PBS for 15–20 minutes at room temperature and immunostained for Vinculin and F-actin as described [16], except that in the case of MDA-MB-231 cells transduced with mCherry or Arl13b-mCherry, permeabilization was performed with PBS with 0.1–0.2% Triton X-100 for 30 minutes and blocking with 0.2% BSA in PBS with 100 mM glycine for 1 hour. Antibodies and concentrations used are listed in Table S2.

4.6. Quantification of Focal Adhesions

For FA quantification, Vinculin-positive FAs located at the ventral side of well spread and isolated cells were quantified using ImageJ software essentially as described by Nardone et al. [37] with the exception that images were not subjected to automatic brightness/contrast and were binarized using MaxEntropy threshold command. Particles were analyzed (size = 0.30–15; circularity = 0.00–0.99) and the size of detected particles was used to calculate FA mean size. Images were acquired using an LSM 710 (Zeiss) confocal microscope equipped with a Plan-Apochromat 63/1.40 Oil Ph3 lens and Zen Blue 2010b SP1software.

4.7. Immunoprecipitation and Immunoblotting

Immunoblotting was essentially performed as described previously [16], except that cells were lysed in cold lysis buffer containing 50 mM Tris-HCl, pH = 7.5; 1% IGEPAL; 150 mM NaCl; 1 mM EDTA; 1mM EGTA; 2 mM $MgCl_2$; 1 mM DTT and 0.1% SDS. Antibodies and concentrations used are listed in Table S2. Uncropped blots are shown in Appendix A.

Cells transduced with lentiviruses encoding mCherry or Arl13b-mCherry were plated in tissue culture dishes coated with 10 µg/mL fibronectin in DMEM with 10% FBS and transfected with integrin β3–EGFP, a kind gift of B. Wehrle-Haller (University of Geneva, Geneva, Switzerland) and Z. Gu (Brigham & Women's Hospital, Harvard Medical School, Boston, State, USA) [28,38] and pEGFP-C3 plasmids using Lipofectamine 2000 (Invitrogen) according to the manufacturer's instructions. Twenty-four hours after transfection, cells were lysed in cold lysis buffer containing 0.1% Triton X-100, in the presence of protease and phosphatase inhibitors for 30 minutes on ice, followed by centrifugation at 21,100 ×*g*, for 30 minutes at 4 °C. Immunoprecipitation was essentially performed as described previously Section 3. Antibodies and concentrations used are listed in Table S2.

Commented [m12]: Please confirm

4.8. Flow Cytometry

Cells grown on wells coated with 10 µg/mL fibronectin in DMEM with 10% FBS were resuspended in flow cytometry buffer (PBS with 1% FBS and 2 mM EDTA), incubated with anti-β3-integrin antibody for 1 hour, washed twice with flow cytometry buffer and incubated with secondary Alexa Fluor 488-conjugated anti-mouse antibody for 30 minutes, at 4 °C. Antibodies and concentrations used are listed in Table S2. To exclude dead cells, samples were incubated with 0.5 µg/mL of propidium iodide (PI, Sigma-Aldrich) just before acquisition. Cells were analyzed using a FACS Canto II (Becton Dickinson, City, State if any, Country) flow cytometer analyzer. In the case of MDA-MB-231 cells transduced with pLenti6-Arl13b-mCherry or mCherry, the analysis was performed in a FACS Aria III High Speed Cell Sorter (Becton-Dickinson) in order to gate and analyze only mCherry-positive cells. Experiments were analyzed using FlowJo software (version 10.1r7).

4.9. Human Tissue Samples

Freshly-collected ductal breast carcinoma samples and paired adjacent parenchyma tissues in TRIzol were obtained during surgery by pathologist of Hospital Beatriz Ângelo (HBA, City, State if any, Country). The study was approved by the ethics committee of HBA (approval #0174). The clinicopathological characteristics of breast carcinomas used in the study are described in Table S3. Frozen breast tumor samples and normal adjacent tissues were obtained from the tumor bank of the Pathology Department of Instituto Português de Oncologia de Lisboa Francisco Gentil (IPOLFG). The study was approved by the ethics committee of the Institute (approval #UIC/1050). The clinicopathological characteristics of breast carcinomas used in the study are described in Table S4. Frozen tissues were reduced to powder with a mortar and pestle in liquid nitrogen and total protein extracts obtained by solubilization in extraction buffer (50 mM Tris-HCl, pH = 7.9; 150 mM NaCl; 1 mM EDTA; 2 mM $MgCl_2$; 0.1% SDS; 0.5% sodium deoxycholate; 1% IGEPAL) in the presence of protease and phosphatase inhibitors, for 1 hour on ice, followed by centrifugation at 21,100 ×*g*, for 20 minutes at 4 °C. Total protein amounts in the supernatants were quantified and analyzed by immunoblotting as described [16].

4.10. Murine Breast Cancer Orthotopic Model

Mouse procedures were approved by the national regulatory authority, DGAV (Ref. # 0421/000/000/2016) and the institutional ethics committee (nº36/2016/CEFCM), under the rules of Federation for Animal Science Associations (FELASA), accomplishing the 3Rs through evidence-based guidelines. Six mice were used in each condition, since the strain is syngeneic. In order to establish breast orthotopic tumors, MDA-MB-231 cells (2×10^6), stably transduced with lentiviruses encoding shRNAs targeting Arl13b (E4 and E6), Arl13b-mCherry or the corresponding controls (Empty, Mission or mCherry, respectively) were washed once with PBS and resuspended in 100 µl PBS for subcutaneous injection into the mammary fat pads of 4–6 weeks old BALB/c-SCID female mice. After 6–8 weeks, primary tumors were surgically excised from anesthetized mice and relapse was allowed for more 4–6 weeks. Primary and secondary tumors were collected from euthanized mice, as well as lungs to score metastases. All tissues were formalin-fixed and paraffin-embedded, cut in 3 µm sections and stained with hematoxylin and eosin. Tumor volume was determined by the

Commented [m13]: Please confirm if this is correct.

following formula: v=(ab2)/2 (a = long diameter of the tumor; b = short diameter of the tumor; and v = volume).

4.11. Histology and Immunohistochemistry

Normal and breast tumor formalin-fixed (10%) and paraffin-embedded samples were selected for tissue microarray construction. Briefly, tissue cylinders with 1.4 mm were punched from tumor areas of each donor tissue block and placed in a recipient paraffin block (tissue microarray), using a manual Tissue Puncher/Arrayer (Beecher Instruments, city, state abbrev if USA, country). Endogenous peroxidase on de-paraffinized sections was blocked with EnVision TM Flex Peroxidase-Blocking reagent (Dako, city, state abbrev if USA, country). Antigen retrieval was performed by microwaving in citrate buffer (pH = 6.0) for 20 minutes. Slides were incubated with protein block serum-free (Dako) for 30 minutes and then with anti-Arl13b antibody (Sigma-Aldrich) in EnVisionTM Flex Antibody Diluent (Dako) for 60 minutes at room temperature. The slides were washed and incubated with EnVision/HRP rabbit (ChemMate Envision Kit, Dako) and developed with 3, 3′diaminobenzidine-hydrochloride (DAB, Dako) and counterstained with Harris hematoxylin. Slides were mounted with Entellan and analyzed by light microscopy.

4.12. Statistical Analysis

Numerical data are presented as mean ± standard deviation (SD). Statistical tests used for comparing different data sets are indicated. GraphPad Prism software (version 5.00, company, city, state abbrev if USA, country) was used to perform the statistical tests.

5. Conclusions

Our results suggest a role for Arl13b in breast cancer progression by coordinating actin remodeling and cell-ECM adhesion during cancer cell migration and invasion. These findings also raise the possibility of targeting Arl13b for therapeutic purposes.

Supplementary Materials: The following are available online at www.mdpi.com/xxx/s1, Figure S1: Arl13b silencing efficiency and effect on cell growth, Figure S2: Arl13b overexpression enhances migration and invasion of breast cancer cells, Figure S3: Arl13b localizes to actin-rich structures in breast cancer cells, Figure S4: Arl13b regulates the expression of Myosin IIA and interacts with it in breast cancer cells, preferentially in the active state, Figure S5: Arl13b expression in normal breast tissue and in breast carcinoma, Table S1: Primer sequences used for RT-qPCR, Table S2: Concentrations and dilutions of antibodies and dyes, Table S3: Clinicopathological characteristics of breast carcinomas, Table S4: Title. Video S1: Arl13b localizes to focal adhesions.

Author Contributions: Conceptualization, C.C., A. Faustino and D.C.B.; Validation, C.C., A. Faustino, T.B., J.S. and A. Félix.; Formal analysis, C.C. and A. Faustino; Investigation, C.C., A. Faustino, F.S., I.F., D.V., A. Ferreira, P.C.; T.B., J.S. and A. Félix; Resources, T.B. and J.S.R.; Writing—original draft preparation, C.C., A. Faustino and D.C.B.; Writing—review and editing, C.C., A. Faustino; I.F., D.V., A. Ferreira, P.C., T.B., J.S., A. Félix and D.C.B.; Visualization, C.C. and A. Faustino; Supervision, T.B., A. Félix and D.C.B.; Project administration, A.Félix. and D.C.B.; Funding acquisition, A. Félix and D.C.B.

Funding: This work was supported by PhD fellowships from Fundação para a Ciência e a Tecnologia (FCT) to A. Faustino, A. Ferreira and P.C. (PD/BD/105898/2014, PD/BD/135506/2018 and PD/BD/128339/2017, respectively), a post-doctoral fellowship from FCT to C.C. (SFRH/BPD/78561/2011), the FCT Investigator Program to D.C.B. (IF/00501/2014/CP1252/CT0001), and grants from FCT (PTDC/BIM-MEC/4905/2014) and iNOVA4Health - UID/Multi/04462/2013, a program financially supported by FCT/ Ministério da Educação e Ciência, through national funds and co-funded by FEDER under the PT2020 Partnership Agreement.

Acknowledgments: We would like to thank the histology, flow cytometry and microscopy facilities of CEDOC, the anatomical pathology service of IPO, F. Janody (i3S, Universidade do Porto, Porto, Portugal) for critical reading of the manuscript and the kind gift of reagent, C. Seixas (CEDOC) for assistance with confocal microscopy, and B. Wehrle-Haller (University of Geneva, Geneva, Switzerland), Z. Gu (Brigham & Women's Hospital, Harvard Medical School, Boston, USA), J. Casanova (University of Virginia School of Medicine, Virginia, USA), C. Guimas Almeida (CEDOC) and K. Kontani (University of Tokyo, Tokyo, Japan) for the kind gift of reagents.

Commented [m14]: Please confirm if this is correct.

Commented [m15]: Please add

Commented [m16]: Please check if the individual contribution of each co-author has been stated.

Conflicts of Interest: The authors declare no conflict of interest.

References

1. Ridley, A.J.; Schwartz, M.A.; Burridge, K.; Firtel, R.A.; Ginsberg, M.H.; Borisy, G.; Parsons, J.T.; Horwitz, A.R. Cell Migration: Integrating Signals from Front to Back. *Science* **2003**, *302* (5651), 1704–1709. doi:10.1126/science.1092053.
2. Lambert, A.W.; Pattabiraman, D.R.; Weinberg, R.A. Emerging Biological Principles of Metastasis. *Cell* **2017**, *168* (4), 670–691. doi:10.1016/j.cell.2016.11.037.
3. Parsons, J.T.; Horwitz, A.R.; Schwartz, M.A. Cell Adhesion: Integrating Cytoskeletal Dynamics and Cellular Tension. *Nat. Rev. Mol. Cell Biol.* **2010**, *11* (9), 633–643. doi:10.1038/nrm2957.
4. Paul, N.R.; Jacquemet, G.; Caswell, P.T. Endocytic Trafficking of Integrins in Cell Migration. *Curr. Biol.* **2015**, *25* (22), R1092-105. doi:10.1016/j.cub.2015.09.049.
5. Zhen, Y.; Stenmark, H. Cellular Functions of Rab GTPases at a Glance. *J. Cell Sci.* **2015**, *128* (17), 3171–3176. doi:10.1242/jcs.166074.
6. Casalou, C.; Faustino, A.; Barral, D.C. Arf Proteins in Cancer Cell Migration. *Small GTPases* **2016**, *7* (4), 270–282. doi:10.1080/21541248.2016.1228792.
7. Kjos, I.; Vestre, K.; Guadagno, N.A.; Borg Distefano, M.; Progida, C. Rab and Arf Proteins at the Crossroad between Membrane Transport and Cytoskeleton Dynamics. *Biochim. Biophys. acta. Mol. cell Res.* **2018**, *1865* (10), 1397–1409. doi:10.1016/j.bbamcr.2018.07.009.
8. Sztul, E.; Chen, P.W.; Casanova, J.E.; Cherfils, J.; Dacks, J.B.; Lambright, D.G.; Lee, F.S.; Randazzo, P.A.; Santy, L.C.; Schürmann, A.; et al. ARF GTPases and their GEFs and GAPs: concepts and challenges. *Mol Biol Cell* **2019**, 30(11), 1249-1271. https://doi: 10.1091/mbc.E18-12-0820.
9. Vitali, T.; Girald-Berlingeri, S.; Randazzo, P.A.; Chen, P.-W. Arf GAPs: A Family of Proteins with Disparate Functions That Converge on a Common Structure, the Integrin Adhesion Complex. *Small GTPases* **2017**, 1–9. doi:10.1080/21541248.2017.1299271.
10. Mao, X.; Fan, C.; Yu, X.; Chen, B.; Jin, F. DDEFL1 Correlated with Rho GTPases Activity in Breast Cancer. *Oncotarget* **2017**, *8* (68), 112487–112497. doi:10.18632/oncotarget.22095.
11. Caspary, T.; Larkins, C.E.; Anderson, K.V. The Graded Response to Sonic Hedgehog Depends on Cilia Architecture. *Dev. Cell* **2007**, *12* (5), 767–778. doi:10.1016/j.devcel.2007.03.004.
12. Larkins, C.E.; Aviles, G.D. G.; East, M.P.; Kahn, R.A.; Caspary, T. Arl13b Regulates Ciliogenesis and the Dynamic Localization of Shh Signaling Proteins. *Mol. Biol. Cell* **2011**, *22* (23), 4694–4703. doi:10.1091/mbc.E10-12-0994.
13. Shao, J.; Xu, L.; Chen, L.; Lu, Q.; Xie, X.; Shi, W.; Xiong, H.; Shi, C.; Huang, X.; Mei, J.; et al. Arl13b Promotes Gastric Tumorigenesis by Regulating Smo Trafficking and Activation of the Hedgehog Signaling Pathway. *Cancer Res.* **2017**, *77* (15), 4000–4013. doi:10.1158/0008-5472.CAN-16-2461.
14. Bay, S.N.; Long, A.B.; Caspary, T. Disruption of the Ciliary GTPase Arl13b Suppresses Sonic Hedgehog Overactivation and Inhibits Medulloblastoma Formation. *Proc. Natl. Acad. Sci. U. S. A.* **2018**, *115* (7), 1570–1575. doi:10.1073/pnas.1706977115.
15. Barral, D.C.; Garg, S.; Casalou, C.; Watts, G.F.M.; Sandoval, J.L.; Ramalho, J.S.; Hsu, V.W.; Brenner, M.B. Arl13b Regulates Endocytic Recycling Traffic. *Proc. Natl. Acad. Sci. USA* **2012**, *109* (52), 21354–21359. doi:10.1073/pnas.1218272110.
16. Casalou, C.; Seixas, C.; Portelinha, A.; Pintado, P.; Barros, M.; Ramalho, J.S.; Lopes, S.S.; Barral, D.C. Arl13b and the Non-Muscle Myosin Heavy Chain IIA Are Required for Circular Dorsal Ruffle Formation and Cell Migration. *J. Cell Sci.* **2014**, *127* (Pt 12), 2709–2722. doi:10.1242/jcs.143446.
17. Bray, F.; Ferlay, J.; Soerjomataram, I.; Siegel, R.L.; Torre, L.A.; Jemal, A. Global Cancer Statistics 2018: GLOBOCAN Estimates of Incidence and Mortality Worldwide for 36 Cancers in 185 Countries. *CA. Cancer J. Clin.* **2018**, *68* (6), 394–424. doi:10.3322/caac.21492.
18. Pruski, M.; Rajnicek, A.; Yang, Z.; Clancy, H.; Ding, Y.-Q.; McCaig, C.D.; Lang, B. The Ciliary GTPase Arl13b Regulates Cell Migration and Cell Cycle Progression. *Cell Adh. Migr.* **2016**, *10* (4), 393–405. doi:10.1080/19336918.2016.1159380.
19. Vicente-Manzanares, M.; Ma, X.; Adelstein, R.S.; Horwitz, A.R. Non-Muscle Myosin II Takes Centre Stage in Cell Adhesion and Migration. *Nat. Rev. Mol. Cell Biol.* **2009**, *10* (11), 778–790. doi:10.1038/nrm2786.

20. Webb, D.J.; Donais, K.; Whitmore, L.A.; Thomas, S.M.; Turner, C.E.; Parsons, J.T.; Horwitz, A. F. FAK-Src Signalling through Paxillin, ERK and MLCK Regulates Adhesion Disassembly. *Nat. Cell Biol.* **2004**, *6* (2), 154–161. doi:10.1038/ncb1094.
21. Zaidel-Bar, R.; Milo, R.; Kam, Z.; Geiger, B.A. Paxillin Tyrosine Phosphorylation Switch Regulates the Assembly and Form of Cell-Matrix Adhesions. *J. Cell Sci.* **2007**, *120* (Pt 1), 137–148. doi:10.1242/jcs.03314.
22. Zaidel-Bar, R.; Ballestrem, C.; Kam, Z.; Geiger, B. Early Molecular Events in the Assembly of Matrix Adhesions at the Leading Edge of Migrating Cells. *J. Cell Sci.* **2003**, *116* (Pt 22), 4605–4613. doi:10.1242/jcs.00792.
23. Wiseman, P.W.; Brown, C.M.; Webb, D.J.; Hebert, B.; Johnson, N.L.; Squier, J.A.; Ellisman, M.H.; Horwitz, A.F. Spatial Mapping of Integrin Interactions and Dynamics during Cell Migration by Image Correlation Microscopy. *J. Cell Sci.* **2004**, *117* (Pt 23), 5521–5534. doi:10.1242/jcs.01416.
24. Dai, X.; Cheng, H.; Bai, Z.; Li, J. Breast Cancer Cell Line Classification and Its Relevance with Breast Tumor Subtyping. *J. Cancer* **2017**, *8* (16), 3131–3141. doi:10.7150/jca.18457.
25. Pellegrin, S.; Mellor, H. Actin Stress Fibres. *J. Cell Sci.* **2007**, *120* (Pt 20), 3491–3499. doi:10.1242/jcs.018473.
26. Tojkander, S.; Gateva, G.; Lappalainen, P. Actin Stress Fibers—Assembly, Dynamics and Biological Roles. *J. Cell Sci.* **2012**, *125* (Pt 8), 1855–1864. doi:10.1242/jcs.098087.
27. Broussard, J.A.; Webb, D.J.; Kaverina, I. Asymmetric Focal Adhesion Disassembly in Motile Cells. *Curr. Opin. Cell Biol.* **2008**, *20* (1), 85–90. doi:10.1016/j.ceb.2007.10.009.
28. Gu, Z.; Noss, E.H.; Hsu, V.W.; Brenner, M.B. Integrins Traffic Rapidly via Circular Dorsal Ruffles and Macropinocytosis during Stimulated Cell Migration. *J. Cell Biol.* **2011**, *193* (1), 61–70. doi:10.1083/jcb.201007003.
29. Hua, K.; Ferland, R.J. Primary Cilia Reconsidered in the Context of Ciliopathies: Extraciliary and Ciliary Functions of Cilia Proteins Converge on a Polarity Theme? *Bioessays* **2018**, *40* (8), e1700132. doi:10.1002/bies.201700132.
30. Antoniades, I.; Stylianou, P.; Skourides, P.A. Making the Connection: Ciliary Adhesion Complexes Anchor Basal Bodies to the Actin Cytoskeleton. *Dev. Cell* **2014**, *28* (1), 70–80. doi:10.1016/j.devcel.2013.12.003.
31. Menzl, I.; Lebeau, L.; Pandey, R.; Hassounah, N.B.; Li, F.W.; Nagle, R.; Weihs, K.; McDermott, K.M. Loss of Primary Cilia Occurs Early in Breast Cancer Development. *Cilia* **2014**, *3*, 7. doi:10.1186/2046-2530-3-7.
32. Nobutani, K.; Shimono, Y.; Yoshida, M.; Mizutani, K.; Minami, A.; Kono, S.; Mukohara, T.; Yamasaki, T.; Itoh, T.; Takao, S.; et al. Absence of Primary Cilia in Cell Cycle-Arrested Human Breast Cancer Cells. *Genes Cells* **2014**, *19* (2), 141–152. doi:10.1111/gtc.12122.
33. Yuan, K.; Frolova, N.; Xie, Y.; Wang, D.; Cook, L.; Kwon, Y.-J.; Steg, A.D.; Serra, R.; Frost, A.R. Primary Cilia Are Decreased in Breast Cancer: Analysis of a Collection of Human Breast Cancer Cell Lines and Tissues. *J. Histochem. Cytochem.* **2010**, *58* (10), 857–870. doi:10.1369/jhc.2010.955856.
34. Wang, R.; Deng, X.; Yuan, C.; Xin, H.; Liu, G.; Zhu, Y.; Jiang, X.; Wang, C. IFT80 Improves Invasion Ability in Gastric Cancer Cell Line via Ift80/P75NGFR/MMP9 Signaling. *Int. J. Mol. Sci.* **2018**, *19* (11). doi:10.3390/ijms19113616.
35. Taylor, M.D.; Liu, L.; Raffel, C.; Hui, C.; Mainprize, T.G.; Zhang, X.; Agatep, R.; Chiappa, S.; Gao, L.; Lowrance, A.; et al. Mutations in SUFU Predispose to Medulloblastoma. *Nat. Genet.* **2002**, *31* (3), 306–310. doi:10.1038/ng916.
36. Tavares, S.; Vieira, A.F.; Taubenberger, A.V.; Araújo, M.; Martins, N.P.; Brás-Pereira, C.; Polónia, A.; Herbig, M.; Barreto, C.; Otto, O.; et al. Actin Stress Fiber Organization Promotes Cell Stiffening and Proliferation of Pre-Invasive Breast Cancer Cells. *Nat. Commun.* **2017**, *8*, 15237. doi:10.1038/ncomms15237.
37. Nardone, G.; Oliver-De La Cruz, J.; Vrbsky, J.; Martini, C.; Pribyl, J.; Skládal, P.; Pešl, M.; Caluori, G.; Pagliari, S.; Martino, F.; et al. YAP Regulates Cell Mechanics by Controlling Focal Adhesion Assembly. *Nat. Commun.* **2017**, *8*, 15321. doi:10.1038/ncomms15321.
38. Ballestrem, C.; Hinz, B.; Imhof, B.A.; Wehrle-Haller, B. Marching at the Front and Dragging behind: Differential AlphaVbeta3-Integrin Turnover Regulates Focal Adhesion Behavior. *J. Cell Biol.* **2001**, *155* (7), 1319–1332. doi:10.1083/jcb.200107107.

© 2019 by the authors. Licensee MDPI, Basel, Switzerland. This article is an open access article distributed under the terms and conditions of the Creative Commons Attribution (CC BY) license (http://creativecommons.org/licenses/by/4.0/).
