# Supplementary material for: MicroRNA-Mediated Metabolic Reprograming in Renal Cancer
Source: Cancers (Basel). 2019 Nov 20;11(12):1825. doi: 10.3390/cancers11121825 (PMC6966432; doi:10.3390/cancers11121825)
Supplement: Supplementary file 1 [file cancers-11-01825-s001.zip › cancers-638193-suppl-final.pdf]

Supplementary Materials: MicroRNA-Mediated Metabolic Reprograming in Renal Cancer

Joanna Bogusławska, Piotr Popławski, Saleh Alseekh, Marta Koblowska, Roksana Iwanicka-Nowicka, Beata Rybicka, Hanna Kędzierska, Katarzyna Głuchowska, Karolina Hanusek, Zbigniew Tański, Alisdair R. Fernie and Agnieszka Piekiełko-Witkowska

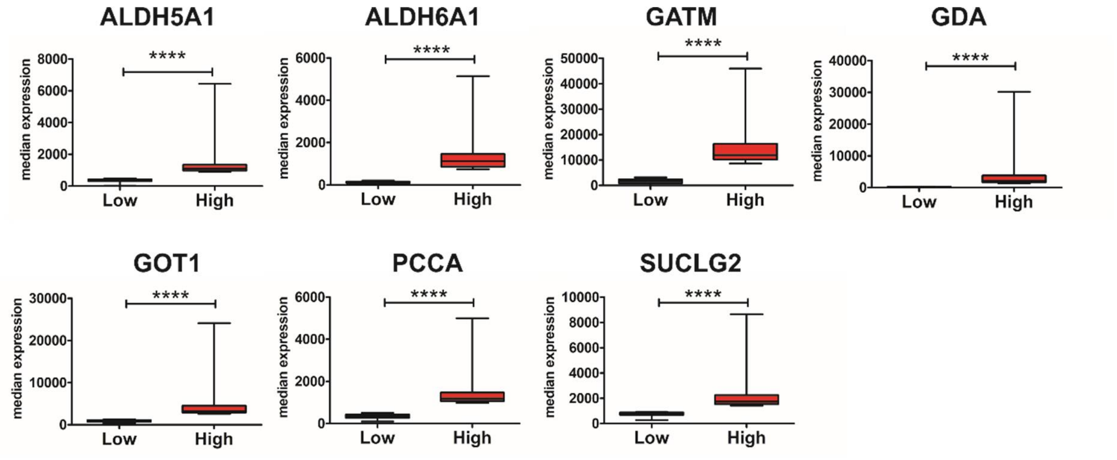

Figure S1. The expression profiles of metabolically relevant genes predicted as targets for microRNAs in two groups of patients stratified into "Low" (n = 130) and "High" (n = 130) expression groups. Transcriptomic data were retrieved from KIRC cohort of TCGA data using OncoLnc tool. Statistical analysis was performed using Mann Whitney test **** $p < 0.0001$. Corresponding K-M plots are given in Figure 1C.

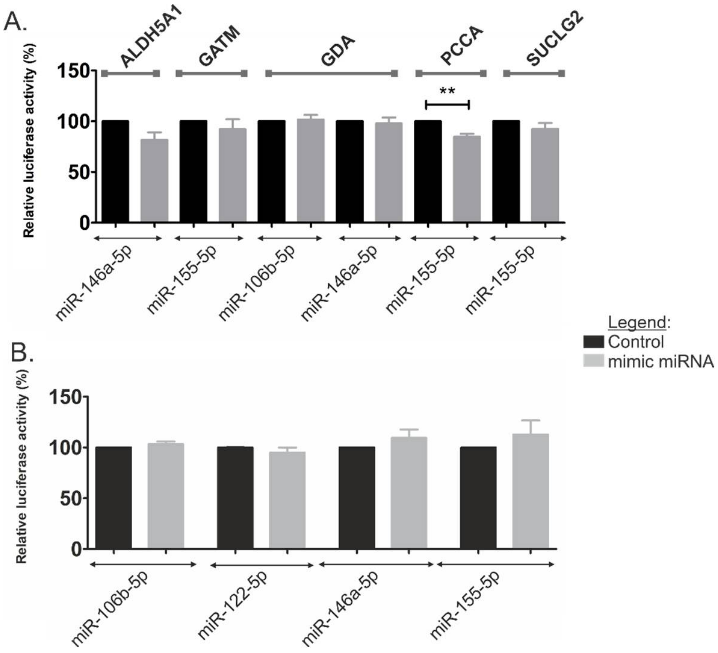

Figure S2. The activity of luciferase reporter system under control of mutated MREs cloned from metabolic genes. (**A**) Caki-2 cells were co-transfected with reporter plasmid bearing mutated MRE for a given microRNA, and either microRNA mimic or non-targeting scrambled control oligonucleotide. (**B**) Caki-2 cells were co-transfected with empty reporter plasmid and either microRNA mimic or non-

targeting scrambled control oligonucleotide. The plots show results of three independent biological experiments. Statistical analysis was performed using *t*-test.

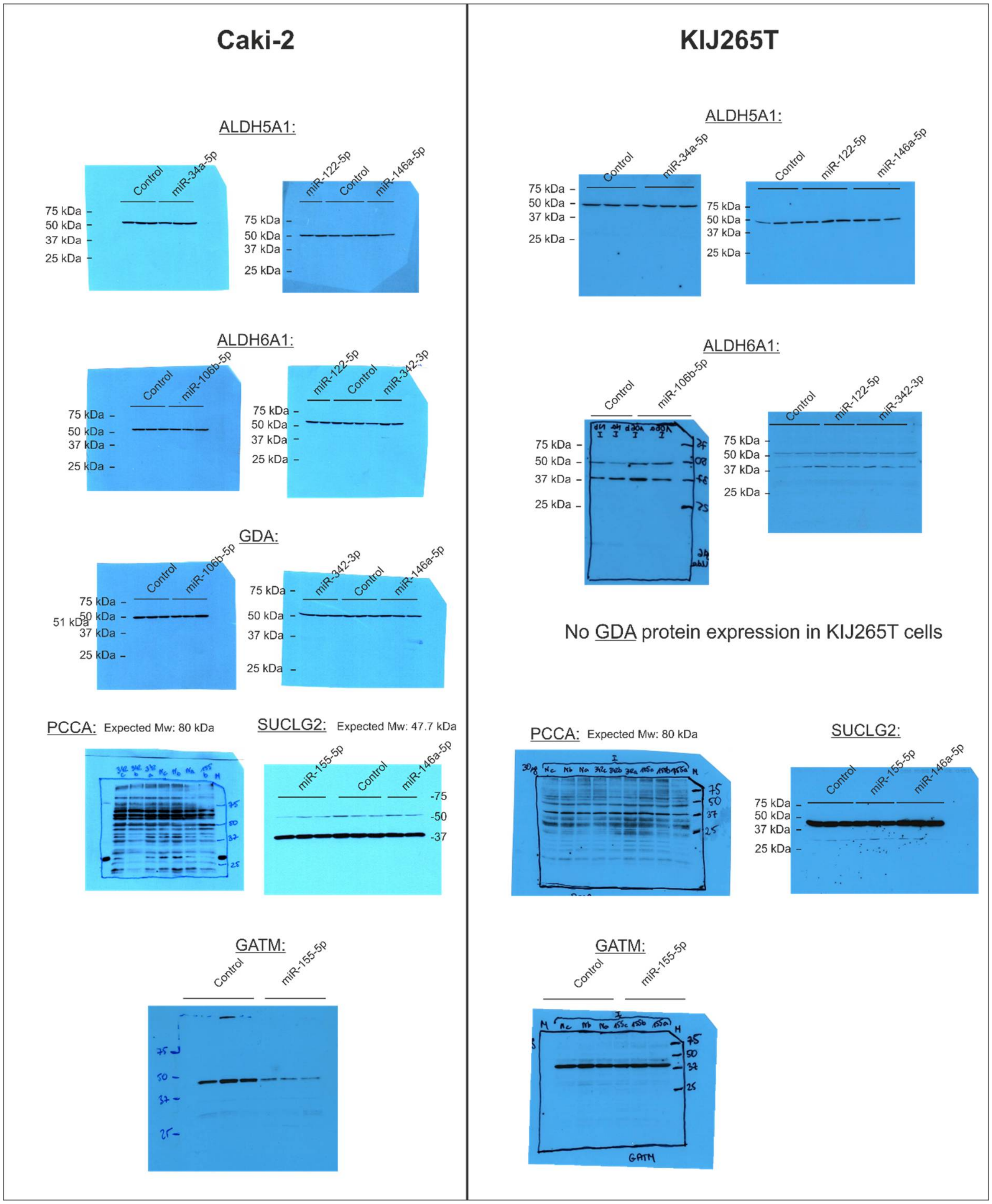

Figure 3. Western blot analysis of proteins encoded by metabolic genes in RCC cells transfected with predicted miRNA mimics or scrambled non-targeting control oligonucleotide. ALDH5A1 (miR-34a-5p, miR-146a-5p, miR-122-5p); ALDH6A1 (miR-106b-5p, miR-122-5p, miR-342-3p); GDA (miR-106b-5p, miR-146a-5p, miR-342-3p); PCCA (miR-155-5p, miR-342-3p) - nonspecific antibody binding; SUCLG2 (miR-146a-5p, miR-155-5p). Representative Western blots of 1 out of at least three (Caki-2) or one (KIJ265T) independent biological experiments are shown.

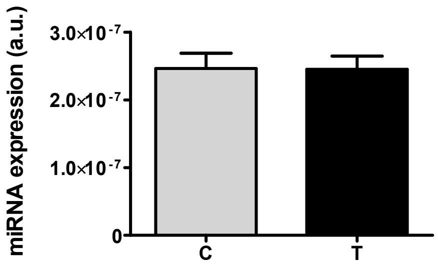

Figure S4. The expression of miR-103a-3p in tissue samples. C: control samples (*n* = 60), T: RCC tumor samples (*n* = 60). Statistical analysis was performed using Wilcoxon matched-pairs signed rank test.

Fils S1. Criteria Used for Selection of miRNAs Targeting Genes Involved in Metabolic Regulation.

Prediction of miRNAs targeting metabolic genes was performed using miRsystem (http://mirsystem.cgm.ntu.edu.tw/). Only microRNA predicted by at least two out of seven predictory algorithms were selected. Next, the list of predicted miRNAs was manually validated using TargetScan (http://www.targetscan.org). Specifically, for each target gene, a list of predicted miRNAs was generated and miRNAs with conserved binding sites in target transcripts were chosen. Next, miRNAs commonly targeting more than one metabolic gene were selected. Finally, published miRNAs, for which published expression data did not fit the profile of predicted target genes were excluded from the analysis.

File S2. The expression of *NFAT5* correlates with expression of the predicted regulatory miRNAs in cancers. The tables show results of analysis performed using StarBase v2.0. (Li et al., 2014) on PanCancer data including transcriptomic data from 14 types of cancers. Statistically significant correlations are highlighted yellow.

**miRNA**	hsa-miR-106b-5p
Target Name	NFAT5(protein_coding)
**Pan-Cancer (14 Cancer Types) miRNA-Target Pearson Correlation Analysis**	

**Cancer Type**	**Sample Num**	**r**	**Rank**	***p*–Value**	**FDR**
Urothelial bladder cancer (BLCA)	229	−0.03252	321375	0.624425	0.7626
Breast cancer (BRCA)	748	−0.29661	20349	1.17376e−16	2.27074e−15
Colon and Rectal adenocarcinoma (CRC)	299	−0.06657	217232	0.251157	0.450663
Glioblastoma multiforme (GBM)	151	−0.18755	57922	0.021111	0.131341
Head and neck squamous cell carcinoma (HNSC)	428	−0.05598	257448	0.247845	0.38168
Chromophobe renal cell carcinoma (KICH)	91	−0.17326	188599	0.100512	0.206942
Clear cell kidney carcinoma (KIRC)	300	−0.44357	9122	6.82632e−16	2.90073e−14
Acute Myeloid Leukemia (LAML)	172	0.00168	360906	0.982525	0.992696
Lung adenocarcinoma (LUAD)	441	−0.12149	112707	0.0106615	0.0370183
Lung squamous cell carcinoma (LUSC)	362	−0.19155	78386	0.000246549	0.00124098
Ovarian serous cystadenocarcinoma (OV)	265	0.27219	5706	6.95172e−06	0.000465882
Cutaneous melanoma (SKCM)	342	0.02575	318932	0.635153	0.783733
Papillary thyroid carcinoma (THCA)	557	0.03567	300243	0.400769	0.52791
Uterine corpus endometrial carcinoma (UCEC)	161	−0.27129	39087	0.000499573	0.00501709

**miRNA**	hsa-miR-155-5p
Target Name	NFAT5(protein_coding)

**Pan-Cancer (14 Cancer Types) miRNA-Target Pearson Correlation Analysis**	**Cancer Type**	**Sample Num**	**r**	**Rank**	***p*−Value**	**FDR**
	Urothelial bladder cancer (BLCA)	229	−0.28397	34636	1.27782e−05	0.000144801
	Breast cancer (BRCA)	748	−0.16677	90392	4.52821e−06	1.97209e−05
	Colon and Rectal adenocarcinoma (CRC)	299	−0.20202	47282	0.00043994	0.00362684
	Glioblastoma multiforme (GBM)	151	0.05083	247447	0.535384	0.779682
	Head and neck squamous cell carcinoma (HNSC)	428	−0.15221	102711	0.00158823	0.00613064
	Chromophobe renal cell carcinoma (KICH)	91	−0.0422	335457	0.691258	0.800155
	Clear cell kidney carcinoma (KIRC)	300	−0.46	7549	4.08978e−17	2.10001e−15
	Acute Myeloid Leukemia (LAML)	172	−0.18435	73154	0.0154804	0.0771633
	Lung adenocarcinoma (LUAD)	441	−0.01352	351778	0.777032	0.864408
	Lung squamous cell carcinoma (LUSC)	362	−0.12184	148019	0.0204111	0.0544061
	Ovarian serous cystadenocarcinoma (OV)	265	0.03661	269645	0.552938	0.784151
	Cutaneous melanoma (SKCM)	342	−0.05015	253316	0.355202	0.551824
	Papillary thyroid carcinoma (THCA)	557	−0.10222	166957	0.0158052	0.0374399
	Uterine corpus endometrial carcinoma (UCEC)	161	−0.2011	78409	0.010528	0.0527066

**miRNA**	hsa-miR-146a-5p
Target Name	NFAT5(protein_coding)

**Pan-Cancer (14 Cancer Types) miRNA-Target Pearson Correlation Analysis**	**Cancer Type**	**Sample Num**	**r**	**rank**	***p*−Value**	**FDR**
	Urothelial bladder cancer (BLCA)	229	−0.09037	209234	0.172916	0.324363
	Breast cancer (BRCA)	748	0.06941	224405	0.0577623	0.101331
	Colon and Rectal adenocarcinoma (CRC)	299	−0.16824	70501	0.00352444	0.0194861
	Glioblastoma multiforme (GBM)	151	−0.02517	303071	0.758994	0.90246
	Head and neck squamous cell carcinoma (HNSC)	428	−0.02965	318848	0.540709	0.67234

	Chromophobe renal cell carcinoma (KICH)	91	−0.26709	113301	0.0104884	0.0359456
	Clear cell kidney carcinoma (KIRC)	300	−0.37605	18950	1.63959e−11	3.3538e−10
	Acute Myeloid Leukemia (LAML)	172	0.40912	3883	2.51551e−08	2.36225e−06
	Lung adenocarcinoma (LUAD)	441	0.06509	215755	0.172402	0.312702
	Lung squamous cell carcinoma (LUSC)	362	−0.03913	300129	0.457904	0.601957
	Ovarian serous cystadenocarcinoma (OV)	265	0.04395	248874	0.476173	0.731646
	Cutaneous melanoma (SKCM)	342	−0.27855	9826	1.63774e−07	6.55928e−06
	Papillary thyroid carcinoma (THCA)	557	−0.10317	165520	0.0148548	0.035494
	Uterine corpus endometrial carcinoma (UCEC)	161	−0.15375	122125	0.0515054	0.165552

**miRNA**	hsa-miR-122-5p					
Target Name	NFAT5(protein_coding)					
**Pan-Cancer (14 Cancer Types) miRNA-Target Pearson Correlation Analysis**	**Cancer Type**	**Sample Num**	**r**	**rank**	***p*−Value**	**FDR**
	Urothelial bladder cancer (BLCA)	229	0.08618	216424	0.193787	0.351437
	Breast cancer (BRCA)	748	−0.0407	286865	0.266205	0.365317
	Colon and Rectal adenocarcinoma (CRC)	299	0.13637	102131	0.0183095	0.0698795
	Glioblastoma multiforme (GBM)	151	0.06283	223168	0.443464	0.716078
	Head and neck squamous cell carcinoma (HNSC)	428	0.0455	280886	0.347737	0.490829
	Chromophobe renal cell carcinoma (KICH)	91	−0.05672	317676	0.593322	0.725232
	Clear cell kidney carcinoma (KIRC)	300	−0.43993	9513	1.2481e−15	5.0856e−14
	Acute Myeloid Leukemia (LAML)	172	0.02716	305725	0.723545	0.862981
	Lung adenocarcinoma (LUAD)	441	−0.05264	245492	0.27002	0.430435
	Lung squamous cell carcinoma (LUSC)	362	−0.05587	262827	0.289075	0.43395
	Ovarian serous cystadenocarcinoma (OV)	265	0.09539	128757	0.121374	0.360471

	Cutaneous melanoma (SKCM)	342	−0.09359	156603	0.0839437	0.210948
	Papillary thyroid carcinoma (THCA)	557	−0.09012	186151	0.0334555	0.0710789
	Uterine corpus endometrial carcinoma (UCEC)	161	0.16439	110948	0.0371719	0.131517

© 2019 by the authors. Licensee MDPI, Basel, Switzerland. This article is an open access article distributed under the terms and conditions of the Creative Commons Attribution (CC BY) license (http://creativecommons.org/licenses/by/4.0/).
